# Intraspecific variation in stomatal architecture, gas exchange, and drought response of a dominant prairie grass sourced from broad climatic gradients

**DOI:** 10.1002/ajb2.70144

**Published:** 2025-12-25

**Authors:** Jack Sytsma, Allison Ricker, Helen Winters, Brian Maricle, Ryann Patterson, Kian Fogarty, Loretta Johnson

**Affiliations:** ^1^ Division of Biology Kansas State University Manhattan KS USA; ^2^ Saginaw Valley State University, University Center MI USA

**Keywords:** *Andropogon gerardi*, C4 grass, common garden, experimental drought, greenhouse, tallgrass prairie

## Abstract

**Premise:**

Understanding how plant populations adapt to water limitation through stomatal traits is key to predicting drought responses. The dominant C_4_ grass *Andropogon gerardi*, distributed across sharp climate gradients in North America, offers an excellent focal species to study stomatal architecture (size and density). Using a common garden, we tested how stomatal architecture relates to home climate, how stomatal architecture influences gas exchange, and how experimental drought affects these responses in a greenhouse. We hypothesized that aridity drives stomatal architecture and that experimental drought reduces the size of stomata but increases their density to maintain photosynthesis.

**Methods:**

We measured stomatal architecture and gas exchange in 25 populations sourced across temperature (4–21°C) and precipitation (350–1400 mm yr⁻¹) gradients under well‐watered conditions. Eight populations (precipitation: 472–1356 mm yr⁻¹) were then subjected to drought (~15% moisture) or were well‐watered (30% control) to assess trait plasticity. Stomatal traits were measured using epidermal peels and light microscopy, gas exchange with a LI‐COR 6400, and network analyses were used to characterize adaptive strategies.

**Results:**

Arid populations exhibited smaller, denser stomata compared to wet populations, and networks demonstrated a trade‐off between stomatal size and density. In the experimental drought, stomatal size decreased. while density increased, with dry populations showing fewer changes than wet populations. Key traits in the network were stomatal size and water‐use efficiency.

**Conclusions:**

*Andropogon gerardi* demonstrated adaptive changes in stomatal architecture. Our findings emphasize the interplay between adaptation and climate, providing important insights into how plants may respond to increased droughts.

Stomata regulate gas exchange by controlling CO₂ diffusion into the leaf and water loss through transpiration (Sack, 1987; Lawson and Mathews, 2020). Stomata control the loss of water vapor by transpiration, so consequently are the driving force for the movement of water throughout the plant (Clark et al., 2022) and ultimately, ecosystem water balance (Berry et al., 2010). Therefore, stomatal regulation remains the primary mechanism for balancing water conservation and carbon gain (Lawson and Vialet‐Chabrand, 2019) and defines the leaf's intrinsic water‐use efficiency (Lawson and Blatt, 2014). Stomata allow plants to optimize carbon gain while minimizing water loss, making them central to plant survival and performance, particularly in water‐limited environments (Clark et al., 2022). Because of this dual role, stomatal architecture (stomatal size and density) is subject to strong environmental selection, particularly along gradients of aridity and drought stress (Hetherington and Woodward, 2003; Buckley, 2019).

A central pattern in stomatal biology is the inverse relationship between stomatal size and density on the leaf, commonly referred to as the stomatal size–density trade‐off (Franks and Beerling, 2009; Lawson and McElwain, 2016). This trade‐off emerges from physical and developmental constraints: As stomata become smaller, more of them can be packed into a given leaf area, increasing maximum photosynthesis, and they tend to open and close more rapidly (Raven, 2014), enabling tighter control of water loss (Martin‐StPaul et al., 2017; Wang et al., 2024). Conversely, larger (but fewer) stomata may incur lower metabolic costs but limit stomatal conductance and photosynthesis (Sack and Buckley, 2016). This size–density scaling relationship has been widely documented across species (Henry et al., 2019; Rahman et al., 2022; Baird et al., 2024; Petrik et al., 2024), but its consistency within species and how it may vary across climate gradients or in response to drought remains poorly understood. If stomatal traits are independently modulated by environmental pressures (e.g., if drought selectively reduces size but not density), the size–density relationship could break down. Alternatively, the relationship may be conserved, suggesting developmental or genetic constraints (Zhang et al., 2012; Cai et al., 2024).

Examining intraspecific variation in stomatal traits can shed light on the balance between stomatal size and density in response to local climate. For example, studies within C_3_ species have shown that plants from xeric environments tend to have smaller and less dense stomata, reducing maximum conductance and transpiration (Bucher et al., 2016; McKown and Bergmann, 2020; Donnelly et al., 2024). However, knowledge on stomatal traits is more limited for C_4_ species. Emerging evidence indicates that within C_4_ grasses, populations from mesic environments often display larger and less‐dense stomata compared to those from arid regions, suggesting that stomatal traits may adaptively vary with water availability (Nunes et al., 2020, 2021; Donnelly et al., 2024).

Major gaps remain in linking climate‐driven trait variation to functional outcomes such as gas exchange and drought tolerance—especially in ecologically dominant C_4_ species. Since stomatal traits directly influence gas exchange and water‐use efficiency (Harrison et al., 2020a), understanding their variation across populations is key to predicting physiological performance under shifting environmental conditions. Moreover, little is known about the plasticity of these traits under drought stress, particularly whether populations from contrasting climates differ in their drought responses when grown in a common environment. This gap limits our ability to separate genetic differentiation from environmental acclimation (Schwinning et al., 2022) and hinders predictions of how such species may respond to ongoing and future drought.

To address these gaps, it is crucial to examine intraspecific variation in stomatal architecture and its effect on gas exchange—particularly intrinsic water‐use efficiency (iWUE) under controlled conditions. While C_4_ plants typically exhibit higher iWUE than C_3_ species (Ellsworth and Cousins, 2016), the extent of intraspecific variation in iWUE across environmental gradients in relation to stomatal architecture remains poorly understood. Our study addresses this gap by investigating variation in stomatal architecture among different C_4_ grass populations and how these populations modulate their stomatal traits in response to experimental drought, highlighting the adaptive value of such trait variation in coping with drought stress (Wang et al., 2019; Westerband et al., 2021).

In this study, we investigated intraspecific variation in stomatal architecture in *Andropogon gerardi* Vitman (big bluestem, Poaceae), a dominant C_4_ grass of North American tallgrass prairies (Knapp et al., 1998). This species spans a wide geographic and climatic range (USDA Plants Database, 2025)—including 4–21°C mean annual temperature and 350–1400 mm yr^–1^ mean annual precipitation (Wang et al., 2016)—making it an excellent system to test how climate shapes stomatal traits and their response to drought stress. We used a common garden greenhouse experiment to test how home climate shapes variation in stomatal traits and influences gas exchange.

Our first objective was to determine whether stomatal architecture is related to climate of origin across all *A. gerardi* populations. We hypothesized that aridity (a driving force of soil moisture availability; Zhang et al., 2023) is the primary driver of stomatal variation, with temperature playing a secondary role. We expected populations from more arid sites to have smaller, denser stomata (Figure 1A, B), a pattern consistent with the stomatal size–density trade‐off because these traits allow for rapid stomatal closure (Martin‐StPaul et al., 2017) and reduced water loss (Shahinnia et al., 2016). In contrast, populations from mesic sites are predicted to have larger, less‐dense stomata, that support higher gas exchange under favorable conditions (Franks and Beerling, 2009). Our second objective was to evaluate whether differences in stomatal architecture are associated with variation in gas exchange. We predicted that higher stomatal density would be correlated with greater photosynthetic rates and stomatal conductance (Figure 1C; Doheny‐Adams et al., 2012), while smaller stomata would be associated with lower transpiration and improved WUE (Figure 1D; Schultz, 2003; Pitaloka et al., 2022).

**Figure 1:**
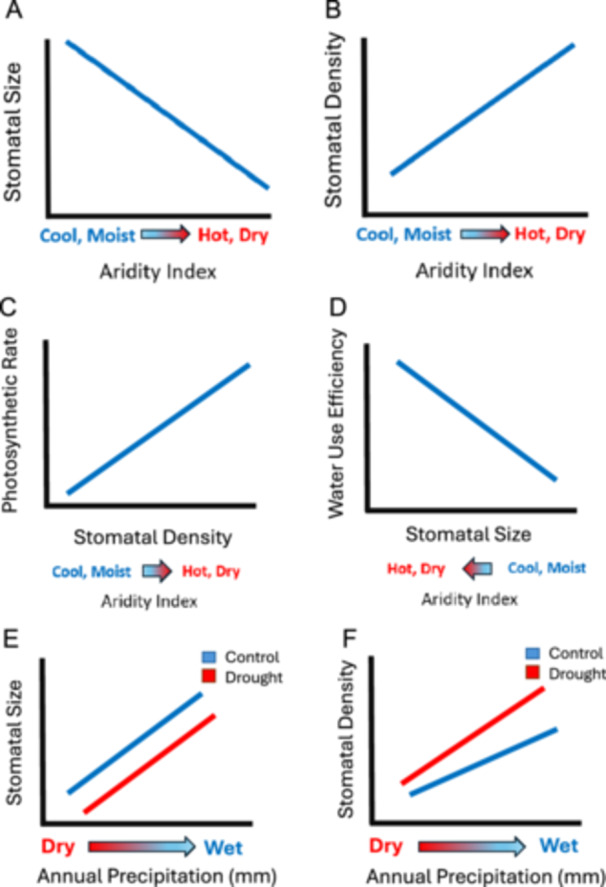
Conceptual models of stomatal architecture across 25 populations of *Andropogon gerardi* regarding (A) stomatal size or (B) density. (C) Models of the relationship between stomatal density and (D) stomatal size and gas exchange (photosynthetic rate and water use efficiency). Conceptual models of the impact of experimental drought on (E) stomatal size and (F) density across a subset of populations across a mean annual precipitation gradient.

Our third objective was to quantify the impact of drought on stomatal architecture and gas exchange using a subset of populations across a precipitation gradient (472–1356 mm yr⁻¹). We hypothesized that experimental drought would induce smaller and more numerous stomata to reduce water loss and maintain photosynthetic function under water stress (Figure 1E, F; Doheny‐Adams et al., 2012). Our final objective was to assess how trait networks reflect variation in stomatal function and gas exchange. We expected that adaptative traits such as stomatal size, density, and conductance would act as “hub” traits (He et al., 2020) that structure plant responses to water availability. Under drought treatment, we further hypothesized that these networks would exhibit increased connectivity (He et al., 2020), reflecting tighter coordination among traits to enhance WUE and minimize water loss.

## MATERIALS AND METHODS

### Study species

Our focal species is *Andropogon gerardi* (big bluestem), which is a dominant species of the U.S. Great Plains and comprises up to 80% of the aboveground biomass (Smith and Knapp, 2003) of tallgrass prairie. This species is a warm season, C_4_, perennial grass with high tolerance for drought (Knapp, 1985), making it a valuable species for studying the impact of climate variability on plant physiology and ecology. Given its role as a foundation prairie grass (Knapp et al., 1998), examining how its stomatal structure influences WUE and gas exchange can provide valuable insights into the mechanisms behind its dominance.

### Experimental design

**Figure 2:**
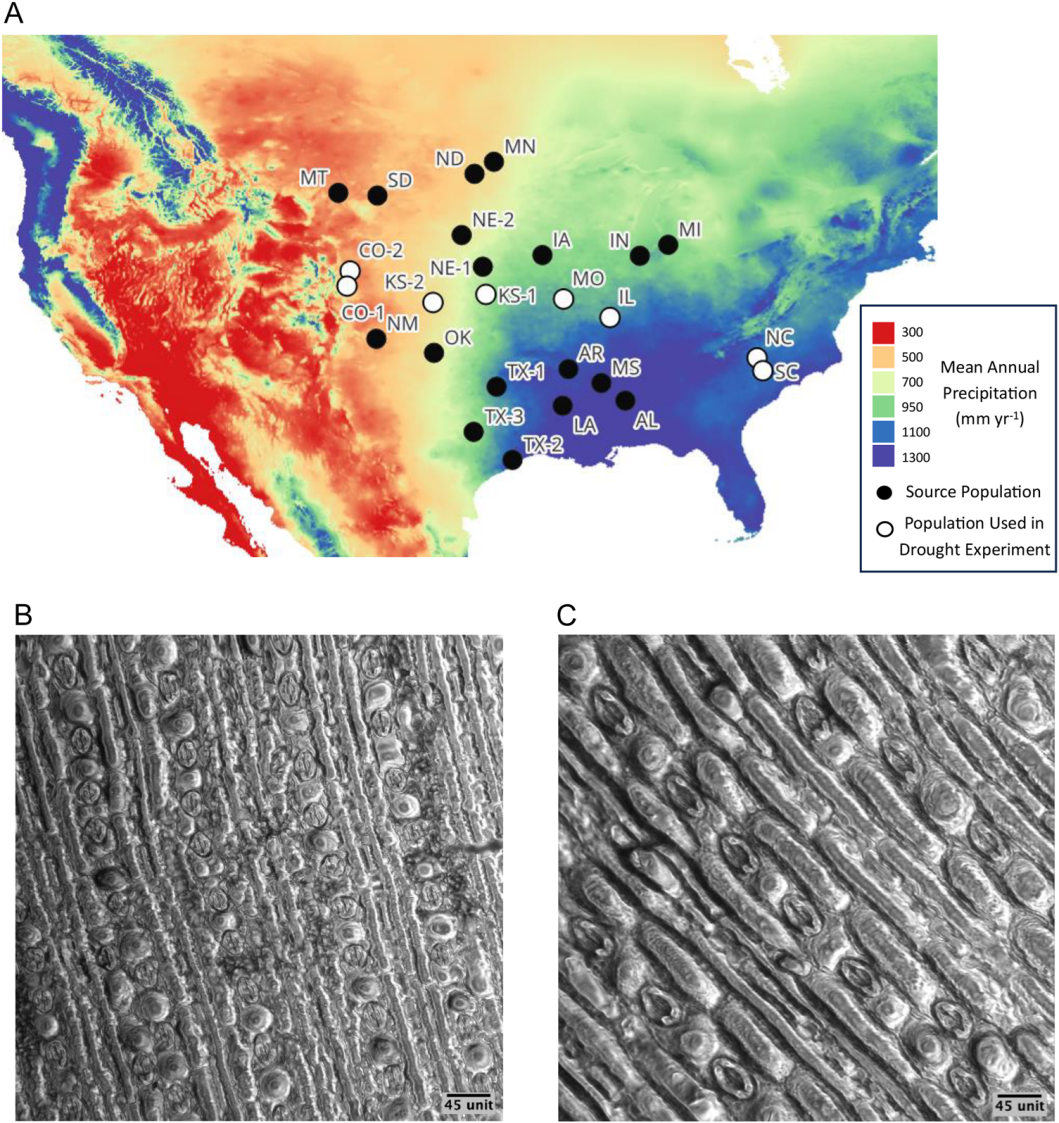
(A) Map of sites of *Andropogon gerardii* from 25 source populations. Points indicate source populations for the main experiment; the color gradient indicates mean annual precipitation (red, low precipitation, dry; blue, high precipitation, wet). Hollow points indicate the subset of eight populations used for the drought experiment. (B) Light micrograph of the surface structure of the abaxial leaf epidermis of CO‐1 and (C) NC, captured at 400× using differential interference contrast optics, highlighting the arrangement of epidermal cells and stomata.

Seeds from 26 populations of *A. gerardi* were collected by hand September–December 2023 from sites located across the range of this species (Figure 2A; Appendix S1: Table S1). Populations were selected from native, unrestored prairies where *A. gerardi* is present across its range. These native prairies were either preserves or private land (not roadside populations). At all sites, permission was granted by the overseeing entity for seed collection. Plants were identified in the field based on key morphological features, including the characteristic three‐branched raceme inflorescence, leaf sheath and ligule traits, using regional floras and identification guides (e.g., *Flora of North America* [Barkworth et al., 2007]). Seeds from individual plants within each source population were pooled to form a single population‐level seed source, which was sown randomly in greenhouse trays to avoid bias in germination or early growth conditions.

In a greenhouse at Kansas State University (Manhattan, KS, USA), we scarified the seed, which were then sown in Berger BM2 potting soil (Berger Horticultural Products, Saint‐Modeste, QC, Canada) in late February 2024 in controlled conditions (25°C day/18°C night). Plants were watered regularly to maintain soil moisture. After 52 days, individual plants were transplanted into pots (20 × 10 × 10 cm) containing Berger BM6 general‐purpose soil mix, then grown with consistent soil moisture, temperature, and light in the greenhouse.

We conducted two experiments. The first, referred to as the main experiment, included all populations of *A. gerardi* (Figure 2A), grown in uniform, well‐watered conditions (~30% soil moisture) to assess baseline trait variation. Each population was represented by six replicates (25 populations × 6 plants per population = 150 plants). For the second greenhouse experiment, we selected a subset of eight populations to represent a broad precipitation gradient in their climates of origin, spanning from Colorado to North Carolina, United States (mean annual precipitation [MAP]: 472–1356 mm yr⁻¹; Figure 2B). This experiment included two soil moisture treatments: control (~30% soil moisture; mean = 31.3%) and drought (~15% soil moisture, mean = 15.4%; Appendix S2: Figure S1B), allowing us to assess population‐level responses to water limitation across populations adapted to contrasting precipitation regimes. The temperature was controlled (mean 25.1°C; range 24–28°C), and light was not supplemented. The drought experiment had eight replicates of each population (8 plants × 2 treatments × 8 populations = 128 plants). Plants were randomized in a block design among greenhouse benches.

Volumetric soil water content was measured daily using METER soil moisture probes (ECH20 10HS Onset S‐SMD‐M005 Large Volume Soil Moisture Sensor, HOBO by Onset Computer Corp., Bourne, ME, USA; 15 cm depth) as volume/volume (%). For the main experiment, one reading was measured per block (six readings per day). Pots were watered daily to field capacity (mean = 31.5%), and the water volume added each day was recorded. Soil moisture throughout the main experiment is reported in Appendix S2: Figure S1A. Total water added in the main experiment was on average 19,150 mL per plant during the 13‐week experiment.

For the drought experiment, we randomly assigned five permanent probes to five control and five experimental drought plants and recorded moisture daily. We also took readings of soil moisture from all plants in the experiment (*N* = 128) weekly. Initially, all pots were watered daily to field capacity, and the volume added was recorded. Starting on Day 63 of the experiment, watering was reduced to once every 2 days for the experimental drought plants. Also in the same greenhouse, control plants were watered daily. By the end of the experiment, 11,775 mL water was added to each control plant, and 6900 mL of water was added to each droughted plant during the 12‐week experiment. Soil moisture throughout the drought experiment is reported in Appendix S2: Figure S1B.

### Response variables

#### Epidermal peels

To assess stomatal size and density, we made epidermal imprints (Figure 2B, C) as described by Pathoumthong et al. (2023) of two leaves on each plant in the main and the drought experiments. Clear nail polish (Hard as Nails by Sally Hansen; Wu and Zhao, 2017) was brushed on the adaxial and the abaxial leaf surfaces, allowed to dry at least 20 min, then peeled off. For the main experiment, we used fresh tissue (live plants in the greenhouse) after plants had sufficient time to reach maturity (2 months after transplanting plants into individual pots). We used two leaves of six replicate plants per population (*N* = 300 leaves).

For the drought experiment, stomatal traits were assessed 6 weeks after drought treatment initiation, allowing plants to acclimate to the water limitation (8 replicate plants × 8 populations × 2 treatments × 2 leaves = 256 leaves). Due to frequent leaf folding in drought‐stressed plants, we standardized the sampling procedure by cutting leaves at the midsection and pressing them flat in a plant press before making the epidermal peels. The same method was also applied to the corresponding control plants in the drought experiment to ensure consistency in sample handling and minimize methodological artefacts. Stomatal traits were directly compared only between control and drought‐treated plants for the drought experiment, not the main experiment.

To analyze the epidermal peels for both experiments, the area of the field of view was calculated from measures with a stage micrometer slide and light microscope (Leica DM750 DIC Microscope) with differential interference contrast optics at 400 × magnification. For both the adaxial and abaxial sides of each plant, we counted three random areas, excluding regions near the main vein (midrib). In the same three fields of view, we measured the size of stomata with an ocular micrometer from one end of the structure where guard cells meet to the same point on the other end of the stomatal complex. The mean of three fields of view for both density (count mm^–2^) and diameter (µm) of stomata was calculated for the abaxial and the adaxial surfaces.

#### Gas exchange

To relate instantaneous gas exchange to stomatal architecture, we measured gas on each plant three times at 1‐month intervals (June, July, August 2024) using a LI‐COR 6400XT infrared gas analyzer (LI‐COR Biosciences, Lincoln, NE, USA) to determine photosynthetic rate, stomatal conductance, transpiration, and internal CO₂ concentration. Intrinsic water‐use efficiency (iWUE) was calculated as photosynthetic rate/stomatal conductance.

Measurements were taken between 10:00 and 14:00 hours on a young, fully expanded leaf per plant. For both experiments, we made gas exchange measurements by block, and all measurements were completed within 3 days to minimize variation in light availability. Chamber conditions were standardized across all measurements: photosynthetically active radiation (PAR) was set at 1500 µmol photons m⁻² s⁻¹, reference CO₂ at 400 ppm, leaf temperature at 28–30°C, and RH between 50–60%. These settings reflect the high light and moderate VPD (vapor pressure deficit) relevant to midday conditions in tallgrass prairie environments.

Before data were logged, leaves were acclimated in the chamber for ~10 min until the net photosynthetic rate remained stable, defined as having a coefficient of variation (CV) < 1% over a 60‐s interval. In practice, the photosynthetic rate typically stabilized within 5–10 min, and we visually monitored stomatal conductance to ensure it showed no further upward trend during this period. Although CV was formally calculated for photosynthetic rate, values for stomatal conductance were checked qualitatively to confirm stability before logging.

#### Source population climate data

Mean (30‐year normal; 1990–2020) climatic variables were downloaded from the ClimateNA version 7.3 database (Centre of Forest Genetic Conservation, Faculty of Forestry, the University of British Columbia, Vancouver, BC, Canada) on 24 July 2024, based on site GPS coordinates where *A. gerardi* was collected. The main experiment sites ranged from 418–1413 mm yr^–1^ historic MAP (Figure 2A) and 5.8–21.2°C historic mean annual temperature (MAT). We assess precipitation and temperature jointly using the aridity index of origin [calculated as (MAT + 10)/MAP × 1000; Wang et al., 2016]. The home sites for the populations from the drought experiment received 472–1356 mm yr^–1^ MAP (Figure 2A, hollow points). The full climatic data set is found in Appendix S1: Table S1.

### Statistical analyses

#### Main experiment

To investigate variation in stomatal architecture in relation to climate, we evaluated all climate variables listed in Appendix S1: Table S1 using both linear and quadratic regression models. Before the analyses, we checked each response variable for normality, and when a variable violated this assumption, we log‐transformed the data to fit a normal distribution. We built linear and quadratic regressions to assess which climate variable best predicted variation in stomatal architecture. We selected the model with the lowest AIC and conducted an analysis of variance (ANOVA) with the fixed effect of population (*P* < 0.05).

To test how stomatal architecture influences gas exchange, we also fit linear and quadratic models with each stomatal trait (density or size) as the predictor and each gas exchange variable as the response variable (photosynthetic rate, transpiration rate, stomatal conductance, intrinsic water use efficiency, internal carbon dioxide). We analyzed differences between stomatal architecture and gas exchange with a one‐way ANOVA. We conducted separate tests for each gas exchange parameter and to avoid model overfitting, we only assessed one stomatal trait at a time. We selected the model with the lowest AIC with a p‐value cut off <0.05.

#### Drought experiment

To assess variation in stomatal architecture and gas exchange under experimental drought, we conducted an analysis of covariance (ANCOVA) with the fixed effects of population and treatment (control or drought) across a precipitation gradient. We then regressed each stomatal trait across the MAP gradient in the control or drought treatment. We conducted separate tests for each gas exchange variable, and to avoid model overfitting, we only assessed one trait at a time.

To assess changes in gas exchange in the drought experiment, we fit linear and quadratic regressions with each stomatal trait (abaxial or adaxial stomatal size or density) as the predictor and each gas exchange variable as the response variable. We compared models with and without the treatment (drought or control) and trait × treatment interaction terms using AIC, retaining models with significant predictors (p < 0.05) to evaluate whether drought modified the relationship between stomatal traits and gas exchange.

#### Ordination analysis

To relate stomatal architecture and gas exchange jointly to climate of origin, we first used a PCA for the main and the drought experiments. Scores from PC axis one were tested with linear regressions using each climate variable (Appendix S1: Table S1) to assess how climate influences stomatal architecture and gas exchange in ordination space. We selected the model with the lowest AIC and *P* < 0.05.

#### Network analysis

To further evaluate coordination among stomatal and physiological traits, we first constructed correlation matrices using pairwise Spearman rank correlation coefficients across all measured traits (stomatal size, stomatal density, photosynthetic rate, transpiration rate, stomatal conductance, water‐use efficiency, and internal CO₂ concentration). We conducted analyses on the main and the drought experiments, and for the drought experiment, these matrices were calculated separately for control and drought treatments to capture treatment‐specific trait associations. The matrices allowed us to identify strongly correlated trait pairs, using a threshold of |*r*| > 0.8 to define biologically meaningful relationships. Significance levels were assessed using p‐values, and only correlations with *P* < 0.05 were retained for further analysis.

We visualized the correlation matrices using the corrplot package in R (Wei and Simko, 2017), which displays the direction (positive or negative) and strength of each correlation. These matrices provided the foundation for our subsequent plant trait network (PTN) analysis. From the filtered correlation matrices, we constructed PTNs by defining traits as nodes and significant correlations as edges. We calculated four core network metrics: edge density (ED), degree (*k*), closeness (*C*), and betweenness (*B*; He et al., 2020). These metrics quantify the overall integration of the network and help identify central “hub” traits that mediate or coordinate physiological responses (He et al., 2020) and under drought treatment.

## RESULTS

### Arid populations had denser, smaller stomata than cool, wet populations

#### Stomatal architecture

**Figure 3:**
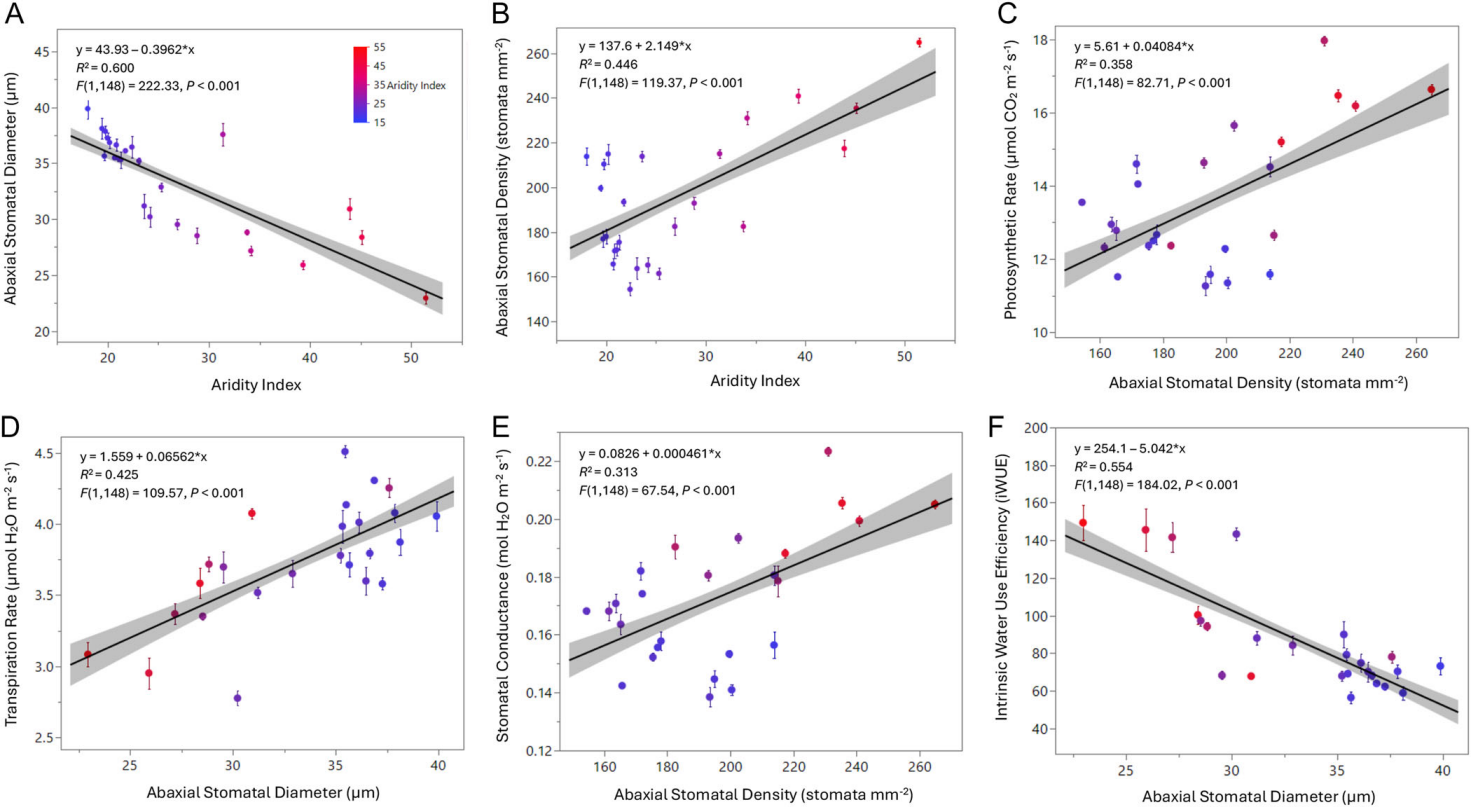
Regressions of abaxial (A) stomatal size and (B) stomatal density in the main experiment of 25 populations. Each trait is regressed across aridity index (climate variable with the lowest AIC). Regressions of (C) photosynthetic rate, (D) transpiration rate, (E) stomatal conductance, and (F) intrinsic water‐use efficiency (iWUE) in the main experiment of 25 populations. Each trait is regressed based on the stomatal trait (abaxial or adaxial stomatal size or density) contributing most to the variation (lowest AIC). The color of the point indicates the aridity index (red,hot, dry; blue, cool, moist). The solid line is the regression, shaded area indicates the confidence interval, points indicate site mean, and error bars indicate site standard error.

Intraspecific variation in stomatal size and density varied significantly with aridity both leaf surfaces, but not with MAP or MAT alone (Appendix S1: Table S2). On the abaxial surface, increasing aridity was associated with smaller stomata (~26 μm in arid regions vs. ~35 μm in cooler, wetter regions; *R*² = 0.60; Figure 3A) and higher stomatal density (~230 stomata mm⁻² in arid vs. ~180 stomata mm⁻² in cooler, wetter regions; *R*² = 0.45; Figure 3B). Leaves were weakly amphistomatous and had an average of 3.90 adaxial stomata mm^−2^, significantly less than the average 201.4 abaxial stomata mm^−2^. Even so, similar trends in stomatal architecture were observed on the adaxial surface as the abaxial surface, where stomata also became denser (~8 stomata mm⁻²; *R*² = 0.59) and smaller (~32 μm; *R*² = 0.63) with increasing aridity, compared to less arid populations (~2 stomata mm⁻²; ~40 μm; Appendix S2: Figure S2A, B). Representative light micrographs of abaxial leaf surfaces and stomatal traits overlaid across a map of North America are provided in Figures S3 and S4.

### Higher stomatal density increases photosynthetic rate, but smaller stomata increase iWUE

Variation in leaf‐level gas exchange was strongly linked to stomatal architecture for all response variables (all *P* < 0.05; Appendix S1: Table S3). For example, photosynthetic rate increased significantly with abaxial stomatal density (*R*² = 0.36; Figure 3C), rising from ~12 μmol CO₂ m⁻² s⁻¹ at lower densities to ~15.5 μmol CO₂ m⁻² s⁻¹ at higher densities (Appendix S1: Table S3). Transpiration rate showed a significant positive relationship with abaxial stomatal diameter (*R*² = 0.43; Figure 3D), ranging from ~3.0 mmol H₂O m⁻² s⁻¹ at smaller diameters to ~4.0 mmol H₂O m⁻² s⁻¹ at larger. Stomatal conductance also increased with stomatal density (*R*² = 0.31; Figure 3E), increasing up to ~0.19 mol H₂O m⁻² s⁻¹ in populations with the highest stomatal density. Conversely, internal CO₂ concentration decreased with increasing stomatal diameter (*R*² = 0.41; Appendix S2: Figure S2C), with values declining from ~195 ppm in populations with smaller stomata to ~190 ppm in those with larger stomata. Abaxial stomatal diameter was the strongest predictor of intrinsic water‐use efficiency (iWUE; Appendix S1: Table S3). Specifically, iWUE was highest (~140 μmol CO₂ m⁻² s⁻¹/mol H₂O m⁻² s⁻¹) at smaller stomatal diameters and lowest (~80 μmol CO₂ m⁻² s⁻¹/mol H₂O m⁻² s⁻¹) at larger sizes (*R*² = 0.55; Figure 3F).

### Experimental drought reduces stomatal size and increases density, but photosynthesis is maintained in dry‐adapted populations

The effects of population were significant in all stomatal traits on both surfaces (p < 0.05 all cases; Appendix S1: Table S4). The interaction of population × experimental drought was significant for all stomatal traits (all *P* < 0.05; Appendix S1: Table S4). Our ANCOVA results confirm the slopes of the regressions of drought and control differed significantly (all *P* < 0.05; Appendix S1: Table S4).

#### Stomatal architecture under experimental drought

**Figure 4:**
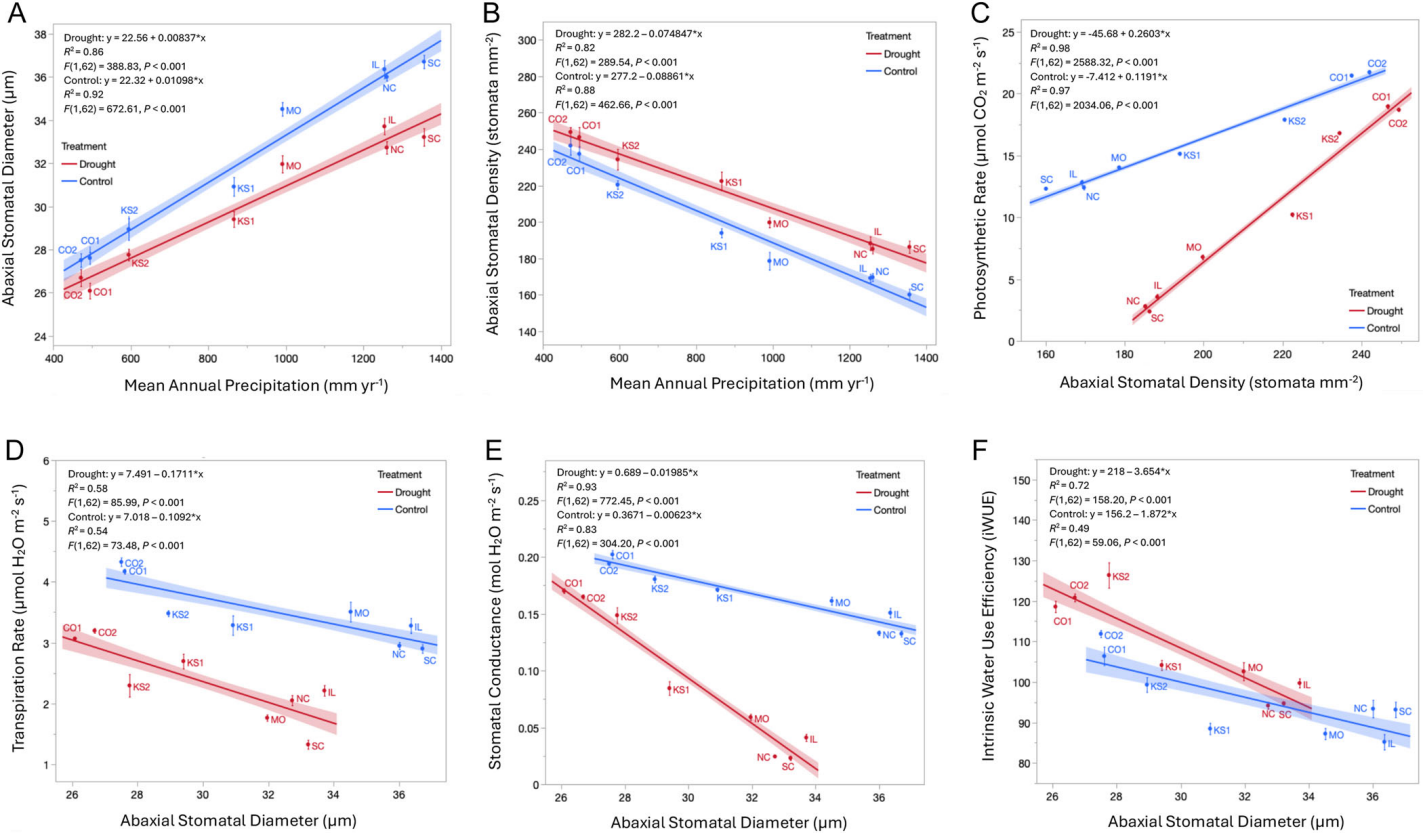
Regressions of (A) stomatal size and (B) stomatal density in the drought experiment of eight populations across the precipitation gradient. Regressions of (C) photosynthetic rate, (D) transpiration rate, (E) stomatal conductance, and (F) intrinsic water‐use efficiency (iWUE) of the drought experiment of eight populations. Each trait is regressed based on the stomatal trait (abaxial or adaxial stomatal size or density) contributing most to the variation (lowest AIC). The red line indicates droughted plants and blue line indicates control plants. The solid line is the regression, shaded area indicates the confidence interval, points indicate site mean, and error bars indicate site standard error.

Stomatal diameter decreased significantly across populations under experimental drought compared to control conditions on both leaf surfaces, with reductions of up to 2.4 μm (Figure 4A). On the abaxial surface, populations originating from sites with higher MAP had a significantly greater decrease in stomatal diameter (~36 μm in control vs. ~32 μm in drought, 12% reduction under drought) than populations from drier origins, which showed minimal change (<1 μm reduction, 3% reduction under drought; Figure 4A). A similar pattern was observed on the adaxial surface, where populations from wetter climates showed a larger decrease in stomatal diameter (~39 μm control vs. ~36 μm drought, 8% reduction under drought), while drier populations exhibited no significant change under drought conditions (Appendix S2: Figure S5A).

In terms of density, abaxial stomatal density significantly increased under experimental drought across all populations, with a minimum increase of 7 stomata mm⁻² (Figure 4B) compared to control. Under drought treatment, populations from drier climates showed a 4% increase in stomatal density (from ~246 to 256 ~stomata mm⁻²), whereas populations from wetter climates showed a 13% increase (from ~163 to ~189 stomata mm⁻²). On the adaxial surface, drought also led to consistent increases in stomatal density (~1.5 stomata mm⁻²; Appendix S2: Figure S5B). These results indicate that drought‐induced variation in stomatal traits is influenced by climate of origin, with more pronounced stomatal shifts occurring in individuals from higher MAP.

#### Gas exchange under experimental drought

In terms of gas exchange under experimental drought, the wet populations showed a significantly greater decline in photosynthetic rate (~60% decline, from ~13 μmol CO_2_ m^–2^ s^–1^ to ~5 μmol CO_2_ m^–2^ s^–1^) than dry populations despite increased stomatal density (Figure 4C; Appendix S1: Table S5). Transpiration rates decreased similarly across populations under experimental drought (~1.0 μmol H_2_O increase across populations; Figure 4D) accompanied by the decrease in stomatal diameter. The wet populations demonstrated significantly greater declines in stomatal conductance (~70% decline, from ~0.15 mmol H_2_O m^–2^ s^–1^ to ~0.05 mmol H_2_O m^–2^ s^–1^; Figure 4E) and internal CO_2_ concentrations (~40% decline, from ~198 μmol CO_2_ m^–2^ s^–1^ to ~192 μmol CO_2_ m^–2^ s^–1^; Appendix S2: Figure S5C) than the dry populations also exposed to experimental drought. Under drought treatment, plants showed a significant increase in iWUE (~20% increase), but only in the dry populations (Figure 4F; Appendix S1: Table S5) due to the decrease in stomatal diameter. Photographs of plants from the drought treatment and controls are presented in Appendix S2: Figure S6.

### Ordination and networks reveal differences in trait integration

#### Ordination of the main experiment

**Figure 5:**
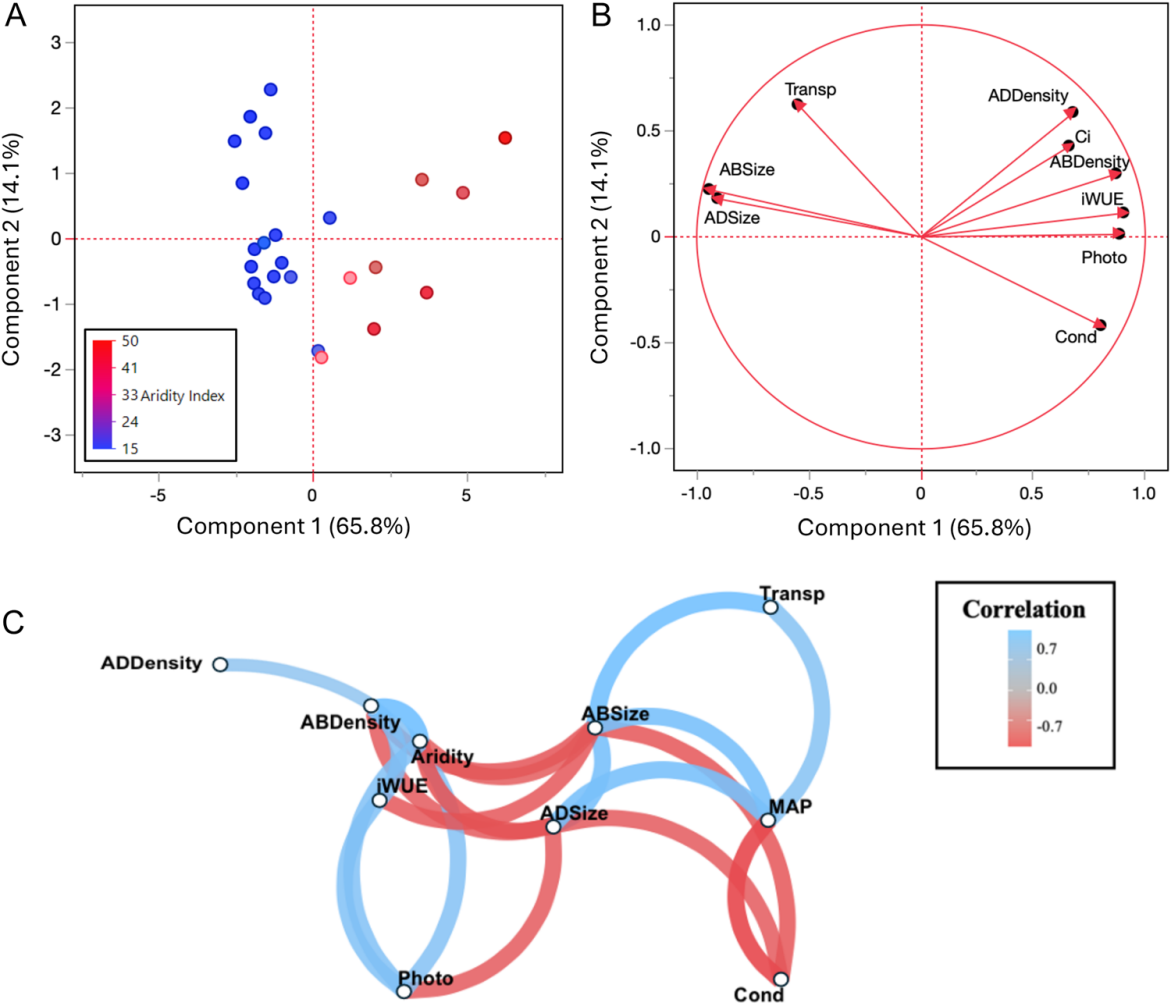
PCA of results for the main experiment including all (A) populations and (B) traits. Points indicate population mean. (C) Plant trait network (PTN) analysis of traits in the main experiment. Network hubs (i.e., hub traits) have the most connections; mediator traits have the shortest path to other traits. Hollow points indicate a variable, the correlation direction is indicated by color (blue, positive; red, negative), and the length of the edge indicates closeness among nodes and shorter lines indicate increased correlation. Variables with no significant correlation (Pearson's –0.7 < *r* < 0.7) do not have an edge drawn and are not shown. Aridity, aridity index; MAP, mean annual precipitation; ABSize, abaxial stomatal diameter, ADSize, adaxial stomatal diameter, ABDensity, abaxial stomatal density; ADDensity, adaxial stomatal density, Photo = photosynthetic rate, Transp = transpiration rate, Cond, stomatal conductance; Ci, internal carbon dioxide concentration; iWUE, intrinsic water‐use efficiency.

In our PCAsfor the main experiment (Figure 5A, B), 65.8% of the variation was explained by component 1 and 14.1% explained by component 2; therefore, we focused mainly on component 1. Regression analysis shows the variance component 1 was explained by aridity index (Appendix S2: Figure S7A). Arid populations were associated with positive values of component 1, and cool, wet populations were associated with negative values of component 1 (Figure 5A). In terms of traits in the PCA (Figure 5B), iWUE, photosynthetic rate, and stomatal density were correlated with positive values on component 1, while transpiration rate and stomatal size correlated with negative values of component 1.

#### Network analysis of the main experiment

**Figure 6:**
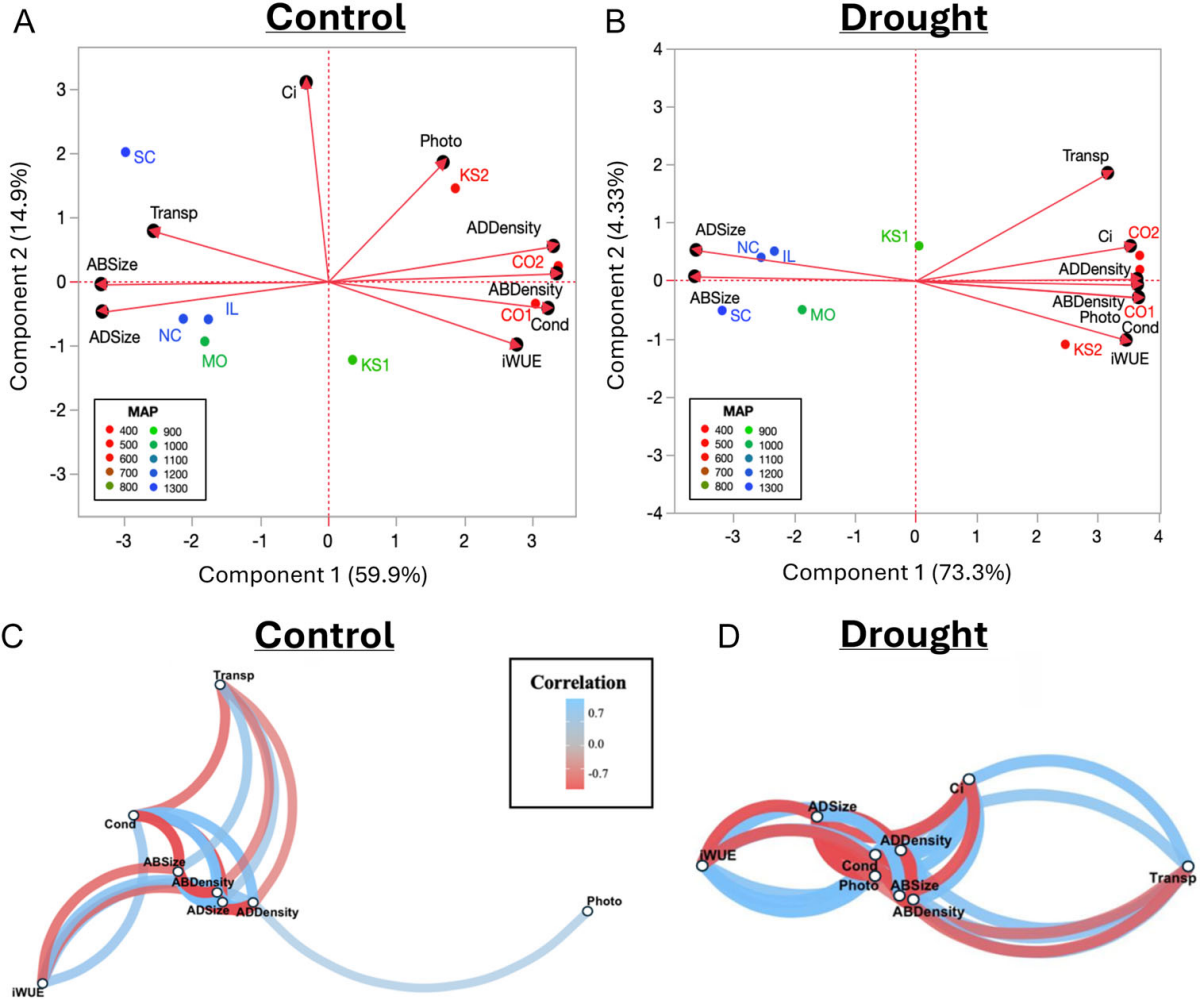
PCA biplot of the subset of eight populations across the precipitation gradient in (A) control and (B) drought treatments. (C, D) Plant trait network (PTN) analysis in drought experiment in (C) control and (D) drought treatments. Network hubs (i.e., hub traits) have the most connections and mediator traits have the shortest path to other traits. Hollow points indicate a variable, the correlation direction is indicated by color (blue, positive; red, negative), and the length of the edge indicates closeness among nodes and shorter lines indicate increased correlation. Variables with no significant correlation (Pearson's –0.7 < *r* < 0.7) do not have an edge drawn and are not shown. Aridity, aridity index; MAP, mean annual precipitation; ABSize, abaxial stomatal diameter, ADSize, adaxial stomatal diameter, ABDensity, abaxial stomatal density; ADDensity, adaxial stomatal density, Photo = photosynthetic rate, Transp = transpiration rate, Cond, stomatal conductance; Ci, internal carbon dioxide concentration; iWUE, intrinsic water‐use efficiency.

Our network analysis (Figure 5C) showed significant correlations among stomatal architecture, physiological traits, and climate of origin. Of the significant relationships, 12 correlations were positive, and 11 correlations were negative (Figure 5C). For key parameters, abaxial stomatal size showed the highest degree (highest *k*‐value; *k* = 7), is the most interconnected, and is thus considered the hub trait (Figure 6A). Aridity index showed the highest closeness (*C* = 0.055) and betweenness (*B* = 6) among all traits and is considered a mediator trait (Figure 5C). These results show that abaxial stomatal size is the most influential trait (hub trait), while aridity index serves as a key mediator trait, indicating its central role in connecting other traits in the network.

#### Ordination of the drought experiment

In control plants of the drought experiment, the PCA (Figure 6A) showed 59.9% of variation was described by component 1 and 14.9% was explained by component 2. Stomatal size and transpiration rate were correlated with negative values of component 1, whereasstomatal conductance, iWUE, and stomatal density were associated with positive values of component 1 (Figure 6A). Regression analysis shows the variance component 1 in the control plants was explained by source population MAP (Appendix S2: Figure S7B).

For the droughted plants in the drought experiment (Figure 6B), 73.3% of the variation was explained by component 1, and 4.33% was explained by component 2. Under experimental drought, all traits but stomatal size were correlated with positive values of component 1 (Figure 6B). Regression analysis shows the variance component 1 in the droughted plants was also explained by source population MAP (Appendix S2: Figure S7C).

#### Network analysis of the drought experiment

Network analysis under control conditions (Figure 6C) revealed significantly lower edge density (ED = 0.20) compared to drought conditions (ED = 0.86), indicating that drought enhances trait connectivity and integration. In well‐watered control plants, abaxial stomatal density emerged as the primary hub trait (highest degree, k = 7), coordinating all traits in the network. Stomatal conductance showed the highest closeness (*C* = 0.04) and betweenness centrality (*B* = 7), identifying it as the key mediator trait. These findings highlight the central role of stomatal traits—particularly stomatal conductance —in coordinating plant function under non‐stressed conditions. Given stomatal conductance's influence on both transpiration and photosynthetic rates, its central position also reinforces its importance in regulating iWUE, even in the absence of drought treatment.

In contrast, under drought treatment (Figure 6D), abaxial stomatal size was considered the hub trait (*k* = 8) coordinating all other traits in the network. Under experimental drought, iWUE was the mediator trait (C = 0.02; B = 8). These results indicate that key traits shift under experimental drought, highlighting the critical roles of stomatal size and WUE in plant response to drought stress. Together, these findings underscored the adaptive shifts in trait interactions in response to drought stress.

## DISCUSSION

In this study, we found clear differences in intraspecific variation in stomatal architecture across populations of *A. gerardi* sourced from across broad (MAP 350–1400 mm yr^–1^, MAT 4–21°C) climate gradients (Appendix S2: Figure S8). To the best of our knowledge, our study is one of the widest ranging of climatic controls on intraspecific variation in stomatal architecture. It is especially relevant in the context of increasing drought (Hetherington and Woodward, 2003; Serna, 2022) because it shows that intraspecific variation in stomatal traits shapes physiological responses to aridity. Given the dominant role of *A. gerardi* in grassland ecosystems, such variation may scale up to influence ecosystem‐level water and carbon fluxes, with important implications for grassland resilience under drought (Berry et al., 2010; Chen et al., 2023).

### Intraspecific variation in the stomatal size–density trade‐off reflects adaptive strategies for water conservation

Our study reveals significant variation in stomatal traits across populations of *A. gerardi*, with clear trends linking stomatal size, density, and area to environmental factors, primarily aridity. Smaller and denser stomata increased with aridity of site of origin, suggesting a strategy to conserve water while maintaining other essential gas exchange processes. This pattern of smaller, denser stomata in arid populations aligns with the idea that water‐limited environments may favor traits that enable more dynamic stomatal control (Haghpanah et al., 2024), such as faster closure and responsiveness (Raven, 2014), rather than simply reducing water loss. While higher stomatal density can increase potential for gas exchange, our data (e.g., Figure 3F) show a positive correlation with iWUE, suggesting that denser stomata may support continued CO₂ uptake even during water limitation (Flexas and Medrano, 2002), possibly through finer regulation at the stomatal level.

Several factors could explain the increase in stomatal density combined with smaller stomata in arid populations. First, higher stomatal density can allow for rapid CO₂ uptake during short‐lived moisture availability. Other studies also suggest that under high vapor pressure deficits, plants may maintain or even increase stomatal density while adjusting other leaf traits (e.g., reducing stomatal conductance) to minimize water loss (Xu and Zhou, 2008; Franks and Beerling, 2009; Carlson et al., 2016; Ameer et al., 2023). Hetherington and Woodward (2003) proposed that species in arid regions have increased stomatal density and smaller individual stomata, thus maintaining total stomatal pore area but limiting excessive water loss. Studies on other C₄ grasses found that in some species, increased aridity correlated with higher stomatal density but smaller stomata, which can improve iWUE compared to C_3_ species (Ozeki et al., 2022). For example, in another dominant C_4_ grass, *Bouteloua gracilis*, plants from arid regions exhibited increased stomatal density, possibly as a mechanism to offset reduced stomatal conductance (Monzón‐Burgos et al., 2023). Further, Ocheltree et al. (2020) examined *Panicum* genotypes from different geographic origins and found that stomatal density increased with growing season temperature and aridity, in line with our results.

In terms of stomatal size, plants sourced from arid environments had smaller stomatal diameters, similar to findings in other studies (Hardy et al., 1995; Liu et al., 2023a; Donnelly et al., 2024; Zhao et al., 2024). A reduction in stomatal size can be attributed to the strategy to optimize gas exchange under water‐limited conditions because a reduction in stomatal area decreases the surface available for water vapor to escape (Ocheltree et al., 2014; Sack and Buckley, 2016). However, despite having a smaller stomatal area on the leaf surface, arid populations compensated by increasing stomatal density, especially on the abaxial side of the leaf, suggesting a functional trade‐off (Liu et al., 2023a) in which arid‐adapted populations maintain the ability to exchange gases efficiently without compromising water conservation.

These differences in stomatal traits along a continuum from arid and non‐arid populations underscore how climate can drive adaptive divergence in water‐use strategies. Given that all populations were grown in a common environment, the observed trait variation reflects underlying genetic differentiation, likely shaped by long‐term climatic selection (de Villemereuil et al., 2016). Stomatal traits, including density and size, are known to be genetically controlled, with studies demonstrating that such traits can vary significantly even within a single species, depending on the climatic conditions of the populations (Franks et al., 2012; Varvel et al., 2018; Clark et al., 2022). Such variation in stomatal architecture supports the idea that historical environmental pressures have contributed to the rise of climate‐adapted stomatal phenotypes (Dittberner et al., 2018; Pérez‐Bueno et al., 2022). Previous work has demonstrated strong intraspecific variation in *A. gerardi* in reciprocal transplant experiments (Galliart et al., 2019, 2020), and our findings extend this to traits linked with water regulation. The persistence of distinct climatic regimes over millennia (Axelrod, 1985) likely provided consistent selection favoring population‐level divergence in stomatal architecture. This evolutionary differentiation may enhance resilience to future climate shifts, particularly in arid environments where efficient water regulation is critical (Navarro et al., 2022).

### Variation in stomatal architecture drives carbon gain and water‐use trade‐offs

Our findings reveal a clear relationship between stomatal architecture and leaf‐level gas exchange in *A. gerardi*. Specifically, photosynthetic rate increased with abaxial stomatal density, with plants exhibiting higher stomatal density showing the highest photosynthetic rates compared to plants with lower stomatal density. This positive correlation between stomatal density and photosynthetic rate suggests that denser stomata may enhance CO₂ uptake capacity (Haworth et al., 2023), thereby supporting higher rates of carbon assimilation under favorable conditions. The increase in stomatal density indicates a structural capacity for greater gas exchange, which could contribute to higher instantaneous carbon gain, particularly when water is not limiting. For instance, several studies found that monocot populations with denser stomata had higher photosynthetic rates (Xu and Zhou, 2008; Pitaloka et al., 2022; Al‐Salman et al., 2023). Others have observed that increased stomatal density led to higher stomatal conductance and CO₂ assimilation rates (Tanaka et al., 2013; Sakoda et al., 2020), thereby enhancing photosynthetic performance.

These findings align with the observed link between larger stomata and increased transpiration rates found in other studies (Kardiman and Raebild, 2018; Hasanuzzaman et al., 2023), emphasizing the importance of stomatal traits in regulating water loss and gas exchange in plants. Our results further support the well‐established trade‐off where smaller stomata, when present in higher densities, can enhance gas exchange efficiency by reducing boundary layer resistance (Taiz et al., 2014). This effect emphasizes the role of stomatal control in regulating transpiration and photosynthesis (Franks and Beerling, 2009), particularly in environments where rapid responses to fluctuating conditions are advantageous. This relationship underscores the trade‐off between gas exchange and water loss because larger stomatal openings facilitate greater water movement through the plant while maintaining gas exchange for photosynthesis.

Water‐use efficiency varied with stomatal traits, where plants with smaller stomata exhibited the highest iWUE, indicating more efficient carbon assimilation per unit water lost. While the direct link between stomatal size and faster closure is better established in the context of fluctuating light (Lawson and Vialet‐Chabrand, 2019), smaller stomata may still indirectly contribute to greater iWUE by allowing finer control over gas exchange, particularly when water is limited. For example, in *Brachypodium distachyon*, stomatal traits exhibit plastic responses to abiotic stress, including drought, with smaller or denser stomata associated with increased iWUE (Nunes et al., 2021). The observed inverse relationship between stomatal size and iWUE in our study suggests that reduced pore size may play a role in limiting transpiration without severely restricting CO₂ uptake (Al‐Salman et al., 2023). This intraspecific variation in stomatal architecture highlights the adaptive potential of stomatal traits in response to environmental stressors such as aridity.

The resulting variation in iWUE further emphasizes the adaptive nature of stomatal architecture, with smaller stomata promoting water conservation while larger stomata may facilitate higher CO₂ uptake but with a higher associated risk of water loss (Appendix S2: Figure S8). These findings highlight the critical role of stomatal architecture in regulating plant gas exchange and water‐use efficiency. Variations in stomatal traits can significantly influence a plant's ability to balance water loss and carbon gain (Bertolino et al., 2019). Our findings therefore have important implications for optimizing crop performance and enhancing plant resilience under changing environmental conditions, such as drought. Specifically, as the frequency of drought intensifies (Chiang et al., 2021; IPCC, 2023; King et al., 2024), understanding and leveraging the adaptive potential of stomatal architecture could be key to safeguarding plant populations in an increasingly unpredictable world.

### Experimental drought‐induced changes in stomatal traits reveal population‐specific responses to water stress

In this study, we showed that experimental drought led to a consistent change in stomatal architecture across populations, with the magnitude of change being dependent on climate of origin. Specifically, we observed a general trend of reduced stomatal size in both arid and mesic populations. This reduction in stomatal size likely represents an adaptive strategy to conserve water by minimizing water loss during periods of drought. Smaller stomata decrease pore size, reducing transpiration to conserve water under drought conditions (Bertolino et al., 2019).

However, while all populations exhibited a decrease in stomatal size under drought, the extent of this change varied. Populations from wet environments showed a more pronounced reduction in stomatal size under drought treatment (12% lower) compared to those from dry sites (3% lower). This response may allow wet populations to adjust their stomatal traits to optimize water conservation while still maintaining sufficient gas exchange for photosynthesis under water‐limited conditions (Chua and Lau, 2024), reflecting adaptive variation to drought. In contrast, dry‐site populations showed minimal change in stomatal size under drought, suggesting that these populations are already adapted to water‐limited conditions (Hereford, 2009), with their stomatal traits being more canalized in response to persistent drought stress.

In addition to changes in stomatal size, we observed an increase in stomatal density across all populations under drought, with wet populations showing the greatest increase (13% increase under drought treatment compared to 2% increase in dry populations). This increase in stomatal density likely compensates for the reduced stomatal size, helping to maintain adequate gas exchange and photosynthesis despite the smaller stomatal aperture (Harrison et al., 2020b). Notably, these coordinated changes reflect the maintenance of the stomatal size–density trade‐off under drought conditions, suggesting that this structural constraint persists even under moisture stress. Other studies suggest that environmental changes modulate the developmental pathways that determine stomatal density (Casson and Gray, 2008; Sack and Buckley, 2016), highlighting the complex interplay between environmental conditions and stomatal development (Hofmann et al., 2025). This interplay underscores the plant's ability to adjust its stomatal architecture in response to drought stress, optimizing WUE and photosynthesis (Appendix S2: Figure S8).

Populations of *A. gerardi* from drier environments exhibited smaller stomata but maintained higher photosynthetic rates under drought compared to populations from wetter regions, suggesting greater efficiency in balancing water conservation with CO₂ uptake. These findings align with previous research showing enhanced photosynthetic performance in arid‐adapted *A. gerardi* ecotypes, even under water stress (Maricle et al., 2017) and point to an adaptive advantage in dry conditions. Our results further show that dry‐adapted populations display more canalized stomatal traits, while populations from wetter climates exhibit greater variation under drought. This variation among populations supports the idea that stomatal architecture changes are an adaptive response to environmental gradients (Chen et al., 2024) and contribute to the overall fitness of the species across diverse habitats.

### Trait coordination highlights adaptive strategies for water conservation

Understanding how plant traits coordinate under different environmental conditions is essential for predicting responses to drought (Martínez‐Vilalta et al., 2023). Our ordination allows for the visualization of trait variation and correlation patterns, whereas the network analysis reveals the strength and structure of trait coordination (He et al., 2020; Liu et al., 2023b). By combining ordination and network analyses, we can better understand how *A. gerardi* modulates key traits to balance carbon assimilation and water conservation in response to water limitation.

Our ordination and network analyses of the main experiment encompassing all populations showed that aridity, not precipitation or temperature alone, is a primary driver of variation in stomatal traits and gas exchange. These results suggest that trait shifts in stomatal architecture and physiology are broadly aligned with gradients of aridity. Consistent with the stomatal size–density trade‐off (Lawson and McElwain, 2016; Rahman et al., 2022), we found that stomatal size and density were always negatively correlated across populations. This anatomical constraint reinforces the idea that plants balance number of stomata and size to optimize gas exchange and water‐use efficiency (Henry et al., 2019) across climatic conditions. These findings underscore the relevance of stomatal traits in mediating plant responses to water availability across climatic gradients, consistent with prior studies demonstrating strong environmental control over stomatal and physiological traits (Liu et al., 2018, 2022).

In the drought experiment, under control conditions, the network was relatively sparse, with hub traits related to carbon gain (stomatal conductance, abaxial stomatal density), suggesting a more resource‐acquisitive strategy (Grime, 1988). In contrast, the drought‐treated network exhibited higher connectivity, with stronger associations among traits like stomatal density and iWUE. This denser network structure may reflect increased coordination aimed at limiting water loss, consistent with previous findings that drought promotes trait convergence and integration to enhance resilience (Freshet et al., 2013; Ni et al., 2024). Notably, the persistent negative correlation between stomatal size and density across populations reinforces the stomatal size–density trade‐off (Lawson and McElwain, 2019), suggesting a conserved developmental constraint that underlies adaptive variation in gas exchange and water‐use efficiency.

Understanding these strategies in grassland species such as *A. gerardi* can inform predictions of plant responses to future climate stressors. Specifically, by linking stomatal traits to environmental conditions and their impact on water use efficiency, we can improve predictions of how grassland species will respond to changing climates (Scoffoni et al., 2017). Future research should explore whether these patterns hold across different species and environmental gradients to better predict how plants will respond to increasing drought (King et al., 2024).

## CONCLUSIONS

Our study highlights the significant role of the stomatal size‐density trade‐off in the adaptation of *A. gerardi* populations to water‐limited environments. This study, which spans a 3‐fold climate gradient, provides a unique and comprehensive system to study the genetic and physiological responses of plants to climatic variability—something that other studies with narrower geographic ranges, cannot reveal. This broad environmental scope allows us to highlight how intraspecific variation is shaped by long‐term climate selection and how populations adapt at both the physiological and genetic levels to their respective environments. Our findings highlight that populations from arid regions exhibit smaller, denser stomata, a characteristic that allows them to conserve water (Bertolino et al., 2019) while maintaining essential gas exchange for photosynthesis. The consistent negative correlation between stomatal size and density across populations reinforces the size–density trade‐off (Lawson and McElwain, 2019), suggesting this trade‐off plays a key role in shaping drought‐related trait evolution. Despite reductions in stomatal size under experimental drought, dry populations showed remarkable resilience in photosynthetic activity, demonstrating an ability to balance water conservation with gas exchange. Overall, our findings underscore the critical role of stomatal architecture in regulating iWUE, offering valuable insights into the adaptive mechanisms in drought response (Bertolino et al., 2019). Since dominant species like *A. gerardi* can strongly influence ecosystem function (Zhang et al., 2025), variation in stomatal traits may contribute to shifts in water and carbon cycling at the ecosystem scale (Berry et al., 2010; Chen et al., 2023). By revealing how stomatal architecture varies across climate gradients and influences drought resilience in a dominant grassland species, our findings offer a valuable foundation for predicting how grassland ecosystems will respond to increasing drought frequency and intensity (King et al., 2024).

## AUTHOR CONTRIBUTIONS

J.S.: writing original draft, supervision, conceptualization, investigation, data curation, formal analysis, methodology. A.R.: writing original draft, investigation, methodology, formal analysis, data curation, software, visualization. H.W.: investigation, review, and editing. B.M.: review, editing. R.P.: investigation, review and editing. K.F.: review, editing, validation. L.J.: conceptualization, methodology, project administration, resources, funding acquisition, review, editing.

## CONFLICT OF INTEREST STATEMENT

The authors have no known competing financial interests or personal relationships that could appear to influence the reported work.

## Supporting information


Appendix S1.
**Table S1.** Site information for source populations of *A. gerardi*.
**Table S2**. Population main effect and results from regression analysis of stomatal architecture in the main experiment of 25 populations using home climate variables as predictors.
**Table S3**. Population main effect and results from regression analysis of gas exchange in the main experiment of 25 populations using stomatal traits as predictors.
**Table S4**. Overall effects and results from regression analysis of stomatal architecture in the drought experiment (subset of 8 populations across a precipitation gradient) using climate variables as predictors.
**Table S5**. Overall effects and results from regression analysis of gas exchange in the drought experiment (subset of 8 populations across a precipitation gradient) using stomatal traits as predictors.

Appendix S2.
**Figure S1**. Regressions of soil moisture (volumetric water content) of the (A) main experiment and (B) drought experiment.
**Figure S2.** Regressions of adaxial (A) stomatal size and (B) density in the main experiment of 25 populations.
**Figure S3.** Light microscopy image of the abaxial leaf epidermis of *A. gerardi* sourced from (A) Minnesota and (B) Texas, USA.
**Figure S4.** Maps of the distribution of abaxial stomatal (A) size and (B) abaxial stomatal density across populations of *A. gerardi* overlaid on a map of North America.
**Figure S5.** Regressions of adaxial (A) stomatal size and (B) stomatal density in the drought experiment of eight populations across a mean annual precipitation gradient. (C) Regression of internal carbon dioxide concentration in the drought experiment.
**Figure S6.** (A) Photograph of plants from populations spanning a mean annual precipitation gradient (495–1360 mm yr^–1^) from Colorado to North Carolina USA, showcasing the variation in plant morphology across different precipitation gradients. (B) Photograph of plants from the drought experiment, showing control and drought treatments in populations from Colorado USA (495 mm yr^–1^) and North Carolina USA (1290 mm yr^‐1^).
**Figure S7.** Regressions of the relationship between component 1 and the climate variable contributing to the most variation explained in the (A) main experiment and drought experiment under (B) control and (C) drought treatments.
**Figure S8.** Conceptual model of how home climate shapes stomatal morphology and drought response in *A. gerardi*.

## Data Availability

All data supporting this study are available in a publicly available Dryad repository (https://doi.org/10.5061/dryad.pg4f4qs3k).
