# Supplementary material for: Intraspecific variation in stomatal architecture, gas exchange, and drought response of a dominant prairie grass sourced from broad climatic gradients
Source: Am J Bot. 2025 Dec 25;113(2):e70144. doi: 10.1002/ajb2.70144 (PMC12918839; doi:10.1002/ajb2.70144)
Supplement: Supplementary file 1 — Appendix S1. Table S1. Site information for source populations of A. gerardi. Table S2. Population main effect and results from regression analysis of stomatal architecture in the main experiment of 25 populations using home climate variables as predictors. Table S3. Population main effect and results from regression analysis of gas exchange in the main experiment of 25 populations using stomatal traits as predictors. Table S4. Overall effects and results from regression analysis of stomatal architecture in the drought experiment (subset of 8 populations across a precipitation gradient) using climate variables as predictors. Table S5. Overall effects and results from regression analysis of gas exchange in the drought experiment (subset of 8 populations across a precipitation gradient) using stomatal traits as predictors. [file AJB2-113-e70144-s002.docx]

**Table S1.** Site information for source populations of *A. gerardi*.

| **Site/ State (USA)** | **County** | **Longitude** | **Latitude** | **Mean Annual Precipitation (mm)** | **Aridity Index** | **Growing Season Precipitation (mm)** | **Mean Annual Temperature (°C)** | **Warmest Month Temperature**  **(°C)** | **Growing Season Temperature**  **(°C)** | **Coldest Month Temperature (°C)** |
| --- | --- | --- | --- | --- | --- | --- | --- | --- | --- | --- |
| **AL** | Dallas | -87.12 | 32.31 | 1422 | 20.2 | 508 | 17.7 | 27.6 | 27.3 | 7.0 |
| **AR** | Lonoke | -91.70 | 34.77 | 1232 | 19.5 | 463 | 16.5 | 27.5 | 21.5 | 4.3 |
| **CO-1** | Boulder | -105.28 | 39.99 | 495 | 45.1 | 139 | 10.8 | 23.2 | 22.6 | 0.5 |
| **CO-2** | Boulder | -105.11 | 39.58 | 467 | 45.0 | 143 | 10.7 | 23.0 | 22.5 | 0.4 |
| **IA** | Jasper | -93.01 | 41.56 | 1022 | 20.9 | 418 | 9.7 | 23.6 | 24.7 | -6.2 |
| **IL** | Williamson | -88.83 | 38.78 | 1254 | 19.9 | 483 | 13.6 | 25.6 | 21.9 | 0.4 |
| **IN** | Lake | -87.45 | 41.52 | 1100 | 19.7 | 400 | 10.5 | 23.5 | 25.2 | -3.8 |
| **KS-1** | Riley | -96.61 | 39.22 | 866 | 23.6 | 325 | 12.6 | 26.1 | 24.6 | -1.7 |
| **KS-2** | Logan | -100.81 | 38.77 | 595 | 34.2 | 228 | 11.9 | 25.8 | 27.6 | -1.2 |
| **LA** | Ouachita Parish | -92.04 | 32.60 | 1467 | 19.8 | 574 | 18.5 | 28.2 | 28.8 | 7.9 |
| **MI** | Kalamazoo | -85.76 | 42.17 | 1131 | 22.4 | 382 | 9.6 | 22.4 | 20.9 | -4.2 |
| **MN** | Clay | -96.14 | 47.10 | 719 | 19.1 | 364 | 5.9 | 21.4 | 21.2 | -8.2 |
| **MO** | Calloway | -91.99 | 38.94 | 991 | 21.1 | 389 | 13 | 25.3 | 24.6 | -0.2 |
| **MS** | Yalobusha | -89.75 | 33.94 | 1513 | 18.0 | 598 | 16.7 | 26.9 | 26.8 | 5.5 |
| **MT** | Rosebud | -105.41 | 45.260 | 418 | 43.9 | 194 | 7.7 | 22.1 | 20.5 | -4.9 |
| **NC** | Cabarrus | -80.51 | 35.44 | 1260 | 20.7 | 497 | 15.6 | 26.3 | 24.8 | 4.9 |
| **ND** | Ransom | -97.30 | 46.39 | 652 | 26.9 | 297 | 5.8 | 21.7 | 21.2 | 2.4 |
| **NE-1** | Lancaster | -96.80 | 40.86 | 882 | 25.3 | 361 | 10.6 | 24.5 | 23.5 | -4.7 |
| **NE-2** | Knox | -98.06 | 42.75 | 652 | 28.8 | 270 | 9.5 | 24.2 | 23.3 | -5.1 |
| **NM** | Union | -104.22 | 36.23 | 444 | 51.4 | 170 | 11.7 | 23.5 | 22.2 | 1.0 |
| **OK** | Beckham | -99.68 | 35.61 | 509 | 39.3 | 240 | 7.7 | 22.4 | 20.8 | 1.2 |
| **SC** | Chesterfield | -80.13 | 34.37 | 1256 | 21.3 | 352 | 16.8 | 26.9 | 25.6 | 5.2 |
| **SD** | Lawrence | -103.07 | 45.10 | 654 | 33.8 | 184 | 14.9 | 27.2 | 26.2 | -2.6 |
| **TX-1** | Fannin | -95.03 | 29.37 | 1181 | 21.8 | 419 | 17.5 | 28.4 | 28.5 | 6.1 |
| **TX-2** | Galveston | -97.34 | 31.06 | 1445 | 31.4 | 569 | 21.2 | 28.7 | 29.5 | 7.3 |
| **TX-3** | Bell | -95.97 | 33.72 | 874 | 24.2 | 384 | 8.6 | 22.3 | 21.2 | 6.6 |

**Table S2.** Population main effect and results from regression analysis of stomatal architecture in the main experiment of 25 populations using home climate variables as predictors.

| **Response variable** | **Main effect: Population** | **Aridity Index** | **Growing Season Precipitation (mm)** | **Mean Annual Precipitation (mm)** | **Mean Annual Temperature (**°C**)** | **Warmest Month Temperature (**°C**)** | **Coldest Month Temperature (**°C**)** | **Growing Season Temperature (**°C**)** | **Latitude** | **Longitude** |
| --- | --- | --- | --- | --- | --- | --- | --- | --- | --- | --- |
| **Stomatal Trait** |  |  |  |  |  |  |  |  |  |  |
| **Abaxial Stomatal Diameter (µm)** | **<0.001** | **<0.001** | **0.01** | **0.01** | 0.08 | **0.04** | **0.001** | 0.33 | 0.22 | 0.33 |
| **Abaxial Stomatal Density (stomata mm⁻²)** | **<0.001** | **<0.001** | 0.10 | **0.03** | 0.08 | **0.01** | 0.10 | 0.24 | 0.77 | 0.12 |
| **Adaxial Stomatal Diameter (µm)** | **<0.001** | **<0.001** | **0.04** | 0.09 | 0.08 | 0.22 | **0.01** | 0.21 | 0.30 | 0.08 |
| **Adaxial Stomatal Density (stomata mm⁻²)** | **<0.001** | **<0.001** | 0.10 | **0.01** | 0.11 | 0.06 | 0.10 | 0.06 | 0.41 | 0.17 |

**Table S3**. Population main effect and results from regression analysis of gas exchange in the main experiment of 25 populations using stomatal traits as predictors.

| **Response Variable** | **Main effect: Population** | **Abaxial Stomatal Density (stomata mm⁻²)** | **Abaxial Stomatal Diameter (µm)** | **Adaxial Stomatal Density (stomata mm⁻²)** | **Adaxial Stomatal Diameter (µm)** |
| --- | --- | --- | --- | --- | --- |
| **Gas Exchange** |  |  |  |  |  |
| **Photosynthetic Rate** (**µmol CO₂ m⁻² s⁻¹)** | **<0.001** | **<0.001** | **0.01** | 0.21 | 0.06 |
| **Transpiration Rate** (**mol H₂O m⁻² s⁻¹)** | **<0.001** | **0.04** | **<0.001** | 0.24 | 0.09 |
| **Stomatal Conductance** (**mol H₂O m⁻² s⁻¹)** | **<0.001** | **<0.001** | **0.01** | 0.07 | 0.11 |
| **Intrinsic Water-Use Efficiency (photosynthetic rate/stomatal conductance)** | **<0.001** | 0.16 | **<0.001** | 0.09 | 0.05 |
| **Internal CO_2_  (µmol CO₂ mol⁻¹)** | **<0.001** | 0.06 | **<0.001** | **0.02** | 0.10 |

| **Response Variable** | **Main effect: Population** | **Main effect: Treatment** | **Treatment x Population Interaction** | **ANCOVA** |
| --- | --- | --- | --- | --- |
| **Stomatal Trait** |  |  |  |  |
| **Abaxial Stomatal Diameter (µm)** | **<0.001** | **<0.001** | **0.003** | **0.001** |
| **Abaxial Stomatal Density (stomata mm⁻²)** | **<0.001** | **<0.001** | **0.04** | **0.03** |
| **Adaxial Stomatal Diameter (µm)** | **<0.001** | **0.001** | **0.04** | **0.02** |
| **Adaxial Stomatal Density (stomata mm⁻²)** | **<0.001** | **0.01** | **0.03** | **0.01** |

**Table S4**. Overall effects and results from regression analysis of stomatal architecture in the drought experiment (subset of eight populations across a precipitation gradient) using climate variables as predictors.

| **Response Variable** | **Main effect: Population** | **Main effect: Treatment** | **Treatment x Population Interaction** | **Abaxial Stomatal Density (stomata mm⁻²)** | **Abaxial Stomatal Diameter (µm)** | **Adaxial Stomatal Density (stomata mm⁻²)** | **Adaxial Stomatal Diameter (µm)** |
| --- | --- | --- | --- | --- | --- | --- | --- |
| **Gas Exchange** |  |  |  |  |  |  |  |
| **Photosynthetic Rate** (**µmol CO₂ m⁻² s⁻¹)** | **<0.001** | **0.001** | **<0.001** | **<0.001** | **0.001** | 0.21 | **0.02** |
| **Transpiration Rate** (**mol H₂O m⁻² s⁻¹)** | **<0.001** | **0.002** | 0.12 | **0.04** | **<0.001** | 0.24 | 0.11 |
| **Stomatal Conductance** (**mol H₂O m⁻² s⁻¹)** | **<0.001** | **<0.001** | **<0.001** | **0.003** | **0.02** | **0.002** | 0.30 |
| **Intrinsic Water Use Efficiency (photosynthetic rate/stomatal conductance)** | **<0.001** | **<0.001** | **<0.001** | 0.16 | **<0.001** | **0.003** | 0.07 |
| **Internal CO_2_  (µmol CO₂ mol⁻¹)** | **<0.001** | **<0.001** | **0.05** | **<0.001** | **<0.001** | **0.04** | 0.13 |

**Table S5**. Overall effects and results from regression analysis of gas exchange in the drought experiment (subset of 8 populations across a precipitation gradient) using stomatal traits as predictors.
