# Supplementary material for: Intraspecific variation in stomatal architecture, gas exchange, and drought response of a dominant prairie grass sourced from broad climatic gradients
Source: Am J Bot. 2025 Dec 25;113(2):e70144. doi: 10.1002/ajb2.70144 (PMC12918839; doi:10.1002/ajb2.70144)
Supplement: Supplementary file 2 — Appendix S2. Figure S1. Regressions of soil moisture (volumetric water content) of the (A) main experiment and (B) drought experiment. Figure S2. Regressions of adaxial (A) stomatal size and (B) density in the main experiment of 25 populations. Figure S3. Light microscopy image of the abaxial leaf epidermis of A. gerardi sourced from (A) Minnesota and (B) Texas, USA. Figure S4. Maps of the distribution of abaxial stomatal (A) size and (B) abaxial stomatal density across populations of A. gerardi overlaid on a map of North America. Figure S5. Regressions of adaxial (A) stomatal size and (B) stomatal density in the drought experiment of eight populations across a mean annual precipitation gradient. (C) Regression of internal carbon dioxide concentration in the drought experiment. Figure S6. (A) Photograph of plants from populations spanning a mean annual precipitation gradient (495–1360 mm yr–1) from Colorado to North Carolina USA, showcasing the variation in plant morphology across different precipitation gradients. (B) Photograph of plants from the drought experiment, showing control and drought treatments in populations from Colorado USA (495 mm yr–1) and North Carolina USA (1290 mm yr‐1). Figure S7. Regressions of the relationship between component 1 and the climate variable contributing to the most variation explained in the (A) main experiment and drought experiment under (B) control and (C) drought treatments. Figure S8. Conceptual model of how home climate shapes stomatal morphology and drought response in A. gerardi. [file AJB2-113-e70144-s001.docx]

**Figure S1**. Regressions of soil moisture (volumetric water content) of the (A) main experiment and (B) drought experiment.

**A.**

**B.**


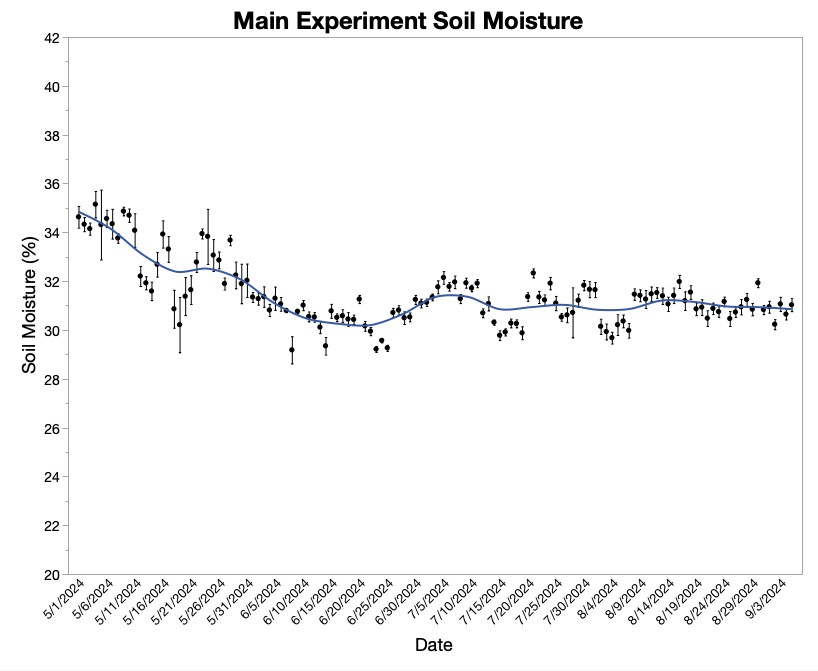


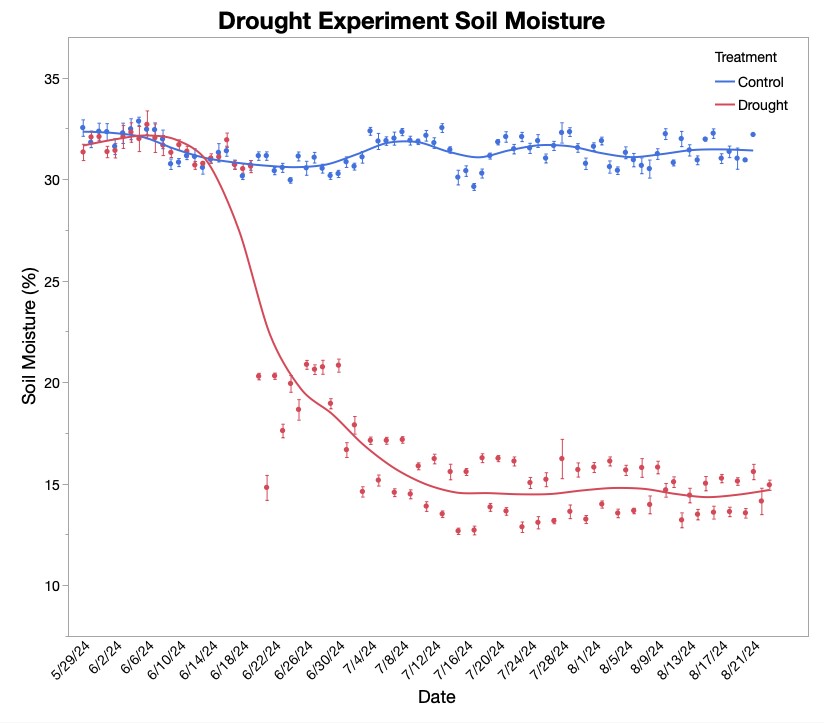


**Figure S2.** Regressions of adaxial (A) stomatal size and (B) density in the main experiment of 25 populations.

**B.**

**A.**


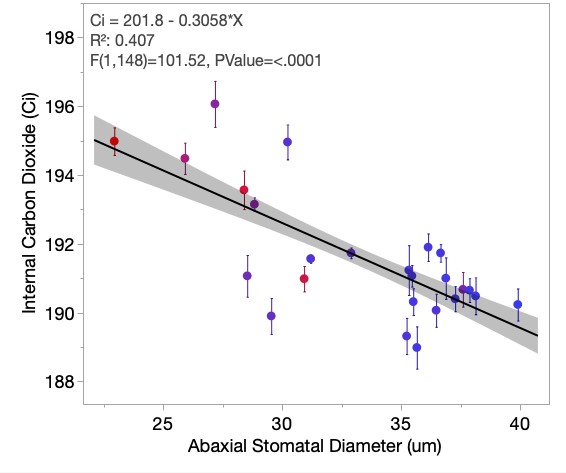

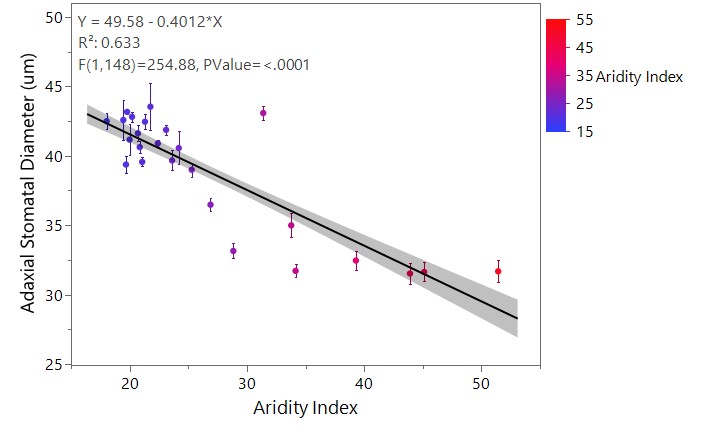

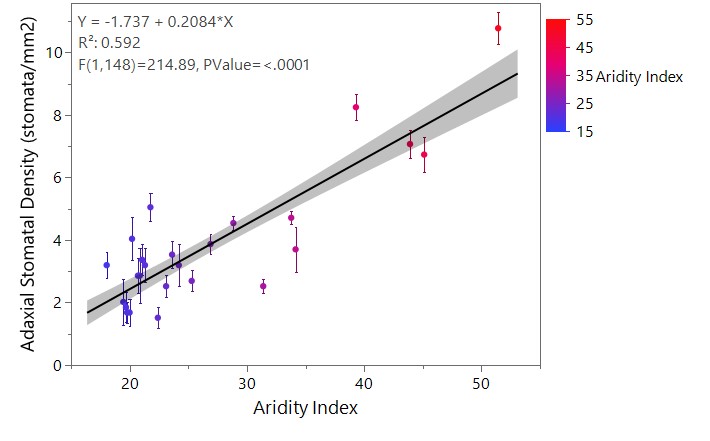


Aridity Index

Aridity Index

Adaxial Stomatal Density (stomata mm^-2^)

Adaxial Stomatal Diameter (**μm)**

Adaxial Stomatal Diameter (**μm)**

Internal Carbon Dioxide (Ci)

y = 49.58 – 0.4012*x

*R*^2^ = 0.633

*F*(1,148) = 254.88, *P* < 0.001

y = -1.737 + 0.2094*x

*R*^2^ = 0.592

*F*(1,148) = 214.89, *P* < 0.001

y = 201.8 – 0.3058*x

*R*^2^ = 0.407

*F*(1,148) = 101.52, *P* < 0.001

**
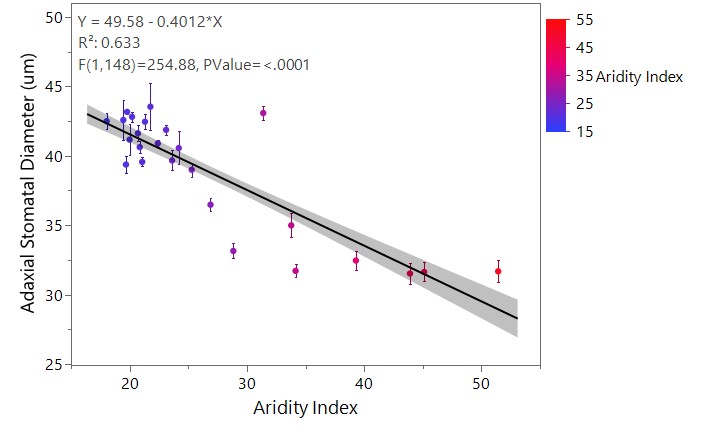
**

**C.**

**Figure S3. Light micrograph of abaxial leaf epidermis of *A. gerardi* sourced from (A) Minnesota and (B) Texas, USA.**

**A.**

**B.**


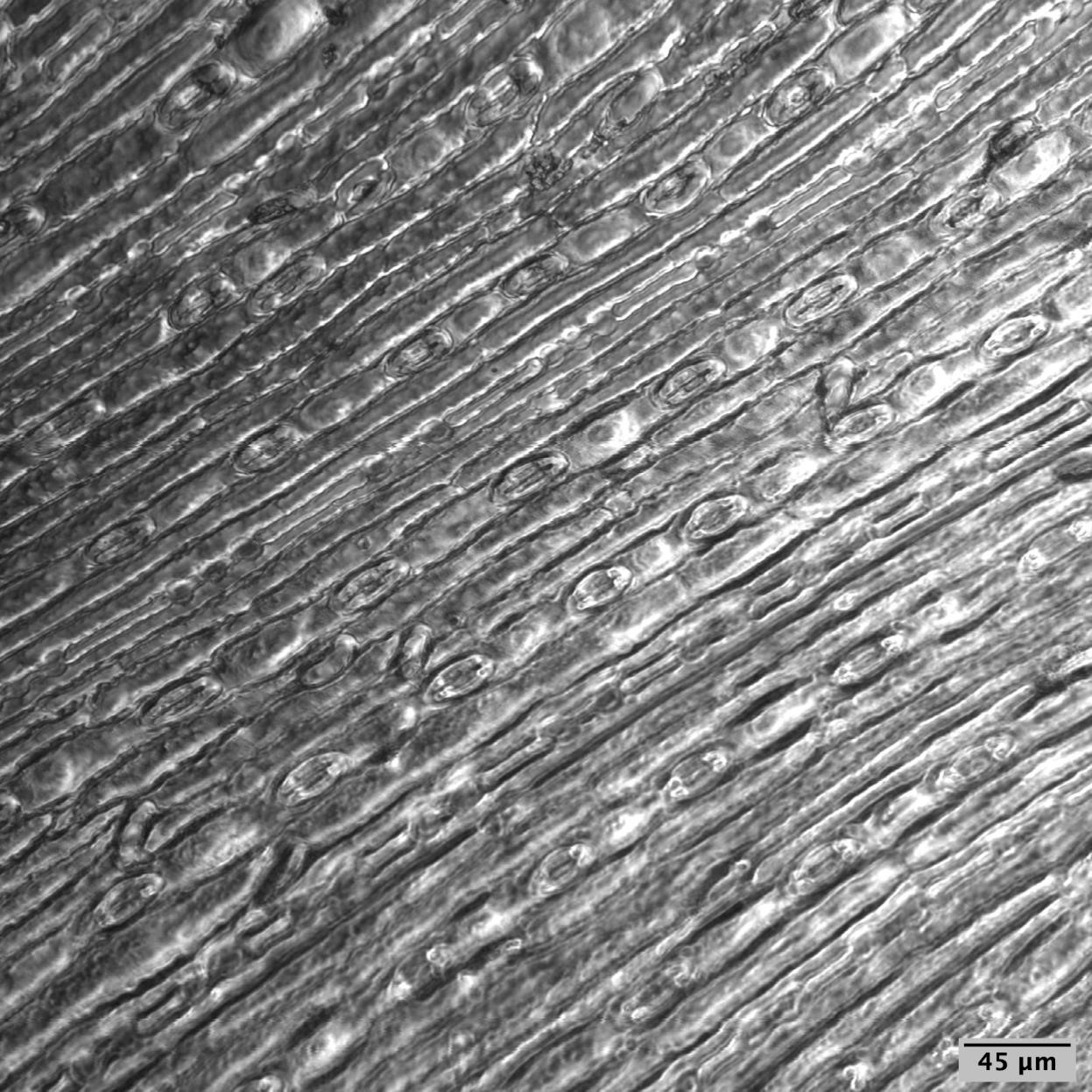

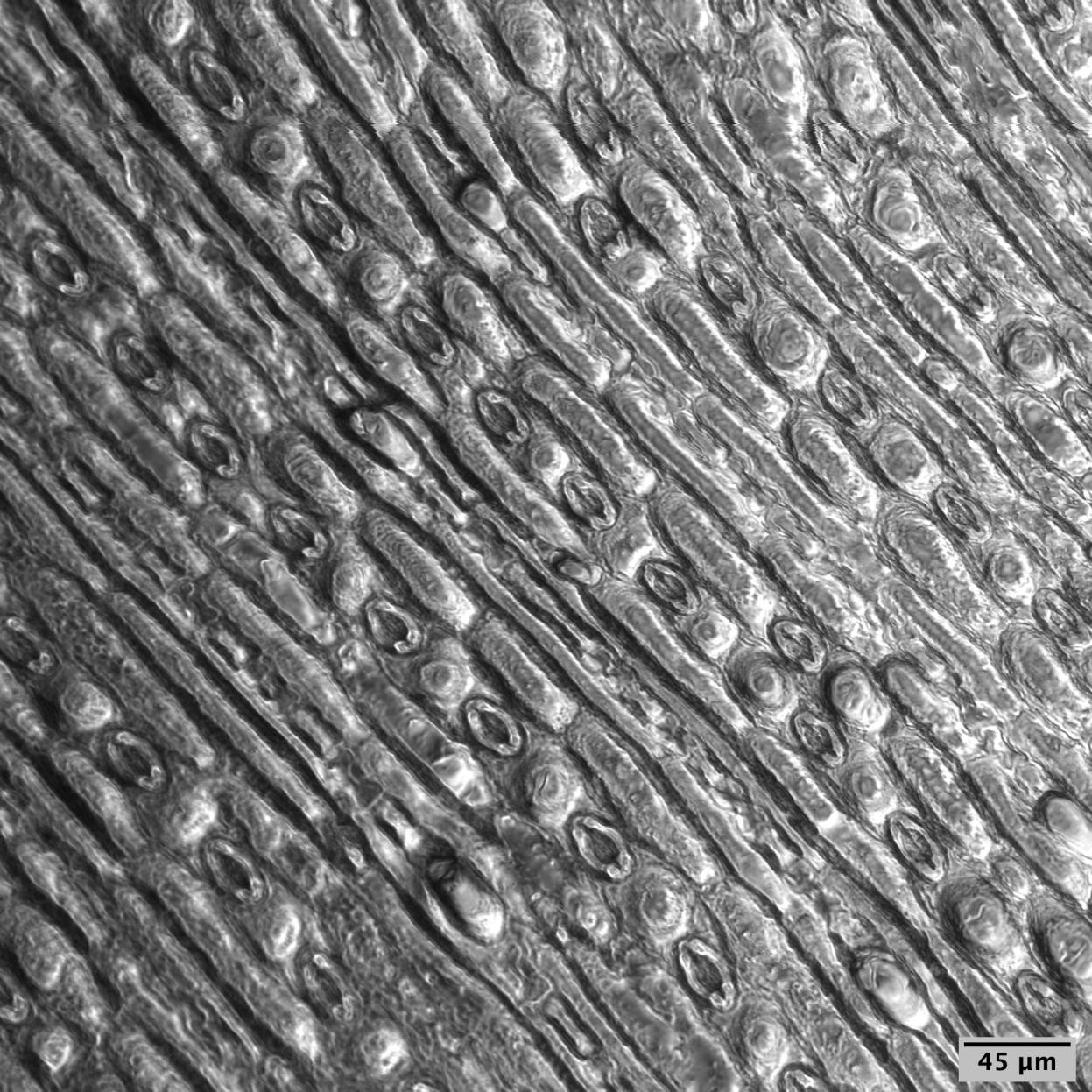


**Figure 4.** Maps of distribution of abaxial stomatal (A) size and (B) density across populations of *A. gerardi* overlaid on a map of North America.


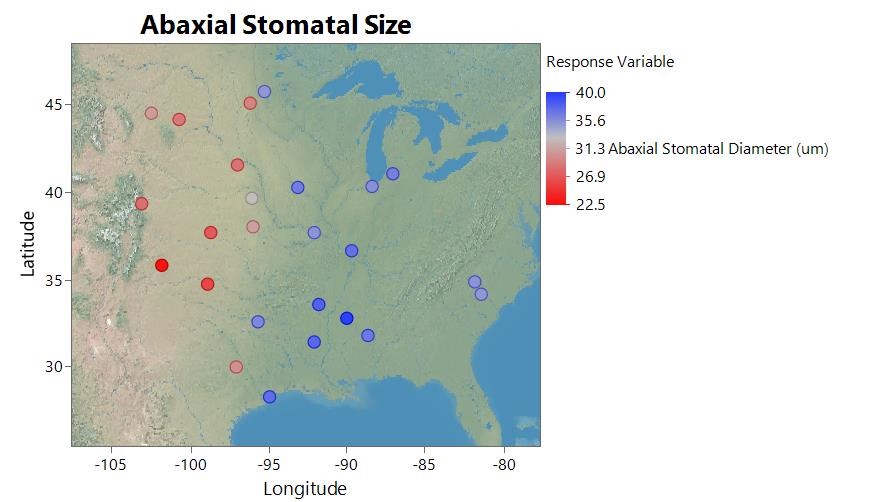

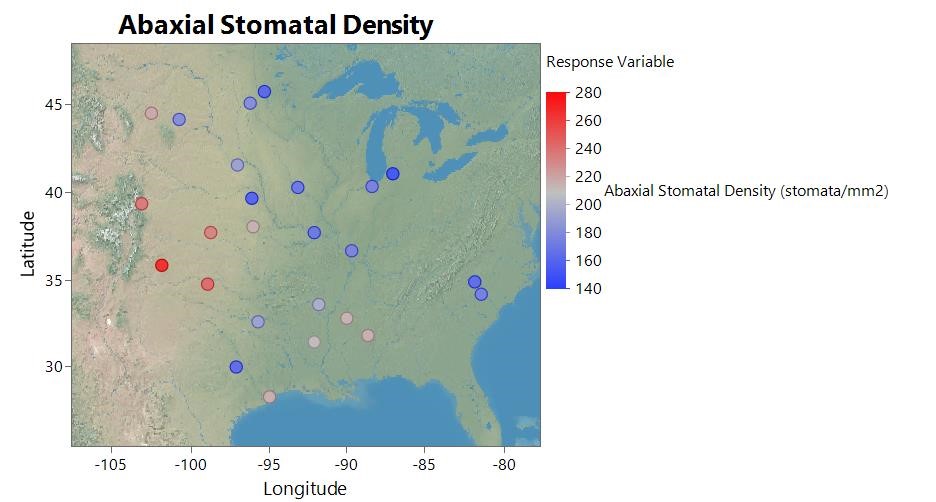


**A.**

**B.**

Abaxial Stomatal Diameter (**μm)**

Abaxial Stomatal Density (stomata mm^-2^**)**

**Figure 5.** Regressions of adaxial stomatal (A) size and (B) density in drought experiment of eight populations across a mean annual precipitation gradient. (C) Regression of internal carbon dioxide concentration in drought experiment.

**A.**

**B.**


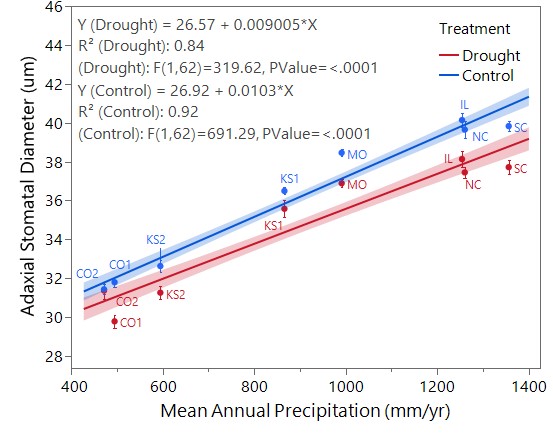

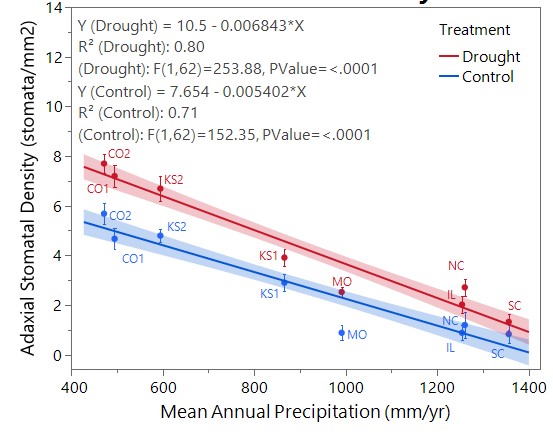

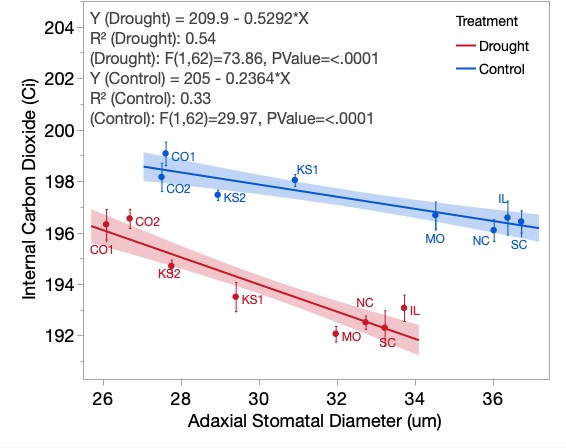


Drought: y = 26.57 + 0.009005*x

*R*^2^ = 0.84

*F*(1,62) = 319.62, *P* < 0.001

Control: y = 26.92 + 0.0103*x

*R*^2^ = 0.92

*F*(1,62) = 691.29, *P* < 0.001

Drought: y = 10.5 – 0.006843*x

*R*^2^ = 0.80

*F*(1,62) = 253.88, *P* < 0.001

Control: y = 7.654 – 0.005402*x

*R*^2^ = 0.71

*F*(1,62) = 152.35, *P* < 0.001

Mean Annual Precipitation (mm yr^-1^)

Adaxial Stomatal Diameter (**μm)**

Mean Annual Precipitation (mm yr^-1^)

Adaxial Stomatal Density (stomata mm^-2^)

Drought: y = 209.9 – 0.5292*x

*R*^2^ = 0.54

*F*(1,62) = 73.86, *P* < 0.001

Control: y = 205.0 – 0.2364*x

*R*^2^ = 0.33

*F*(1,62) = 29.97, *P* < 0.001

Adaxial Stomatal Diameter (**μm)**

Internal Carbon Dioxide (Ci)

**C.**

**Figure S6.** (A) Photograph of plants from populations spanning a mean annual precipitation gradient (495–1360 mm yr^-1^) from Colorado to North Carolina USA, showcasing the variation in plant morphology across different precipitation gradients. (B) Photograph of plants from the drought experiment, showing control and drought treatments in populations from Colorado USA (495 mm yr^-1^) and North Carolina USA (1290 mm yr^-1^).

**A.**

**C.**


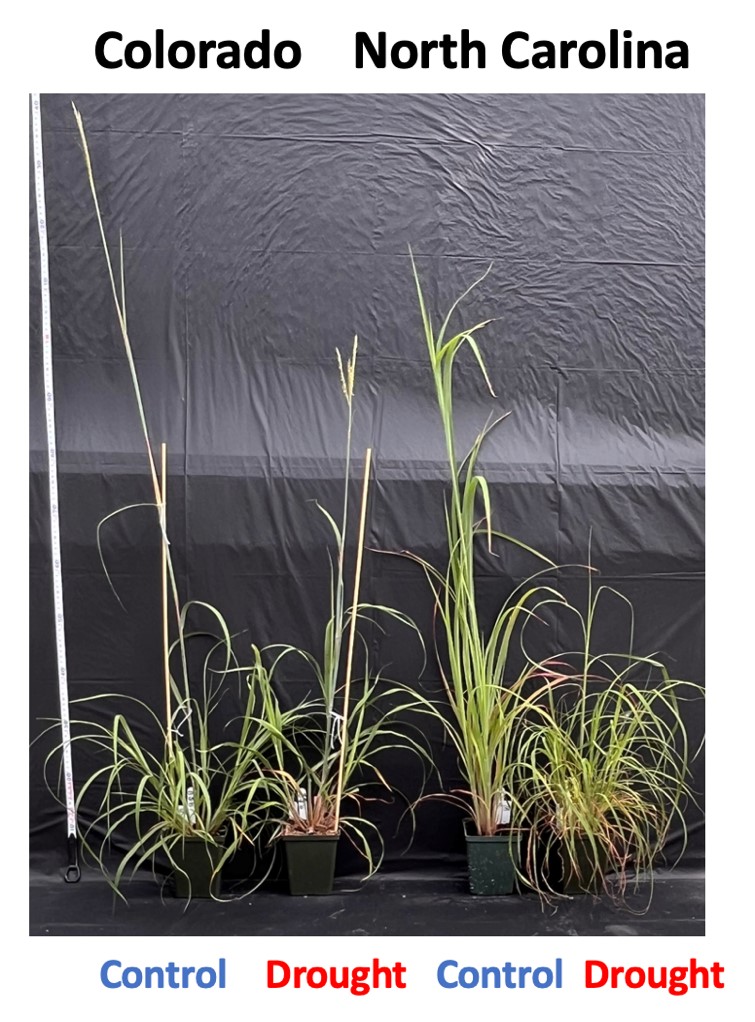


**B.**


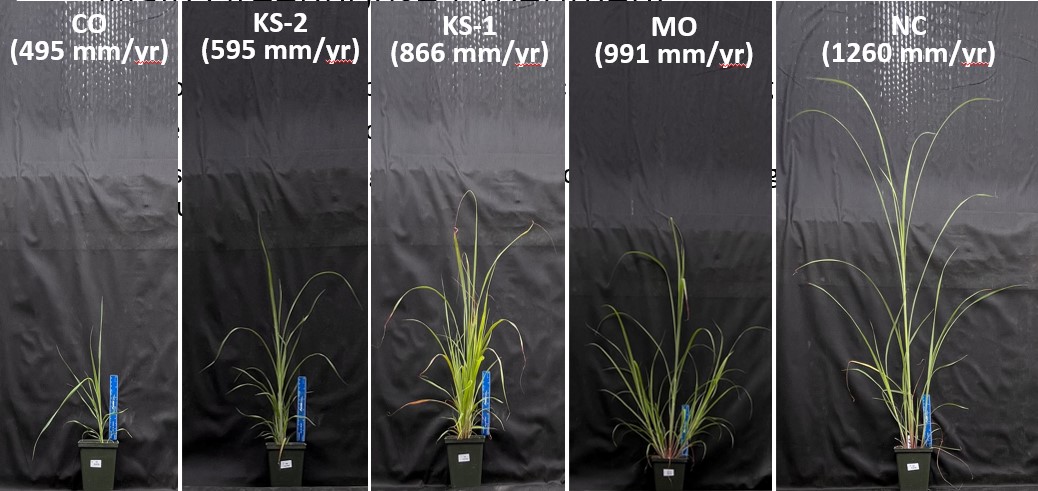


**Figure S7.** Regressions of relationship between component 1 and the climate variable contributing to the most variation explained in the (A) main experiment and drought experiment under (B) control and (C) drought treatments.

**B.**

**A.**


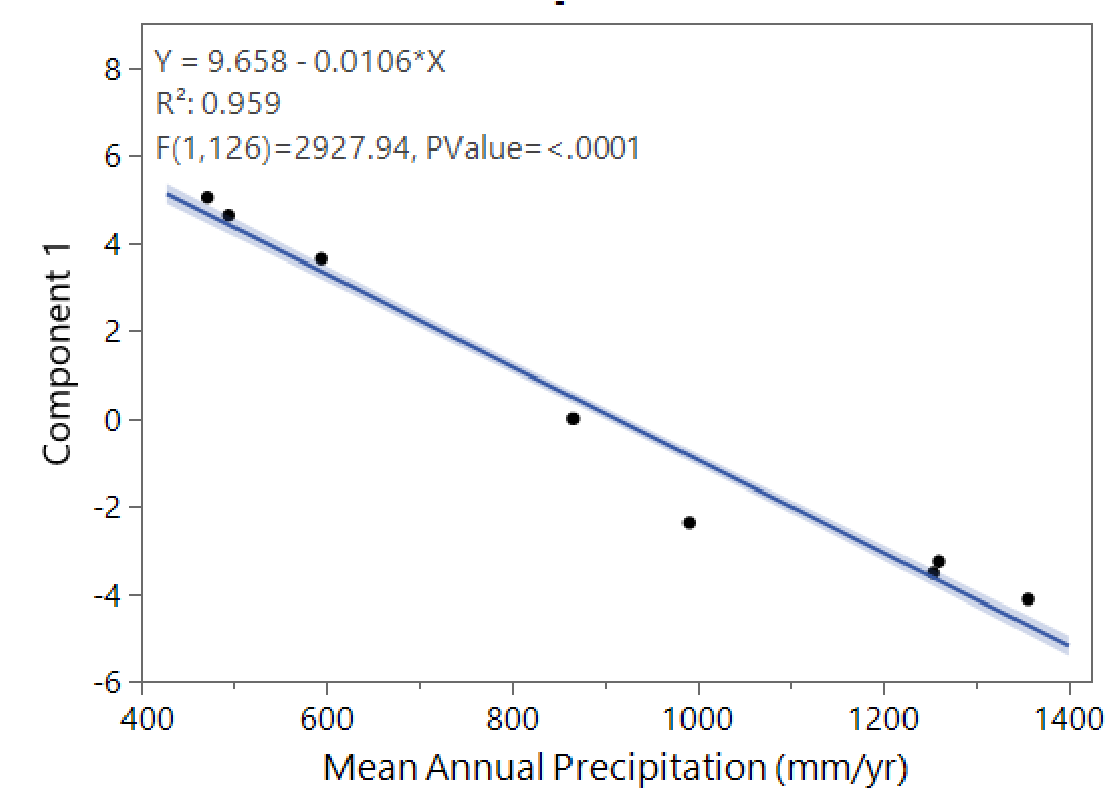

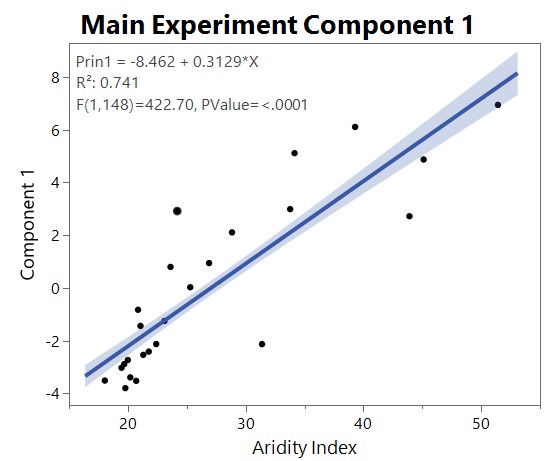

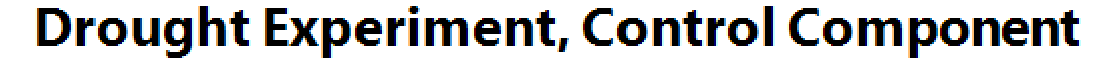

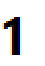

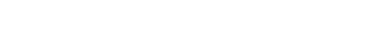


PCA Component 1


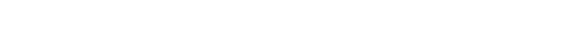


PCA Component 1


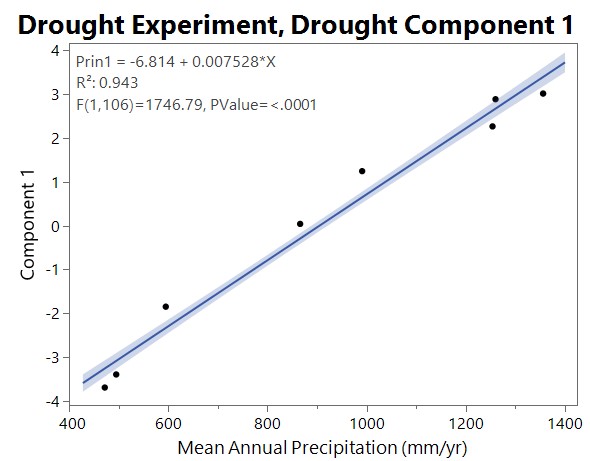

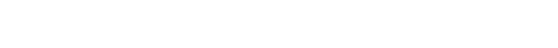


PCA Component 1

Mean Annual Precipitation (mm yr^-1^)

Aridity Index

Mean Annual Precipitation (mm yr^-1^)

y = -8.462 + 0.3129*x

*R*^2^ = 0.741

*F*(1,148) = 442.70, *P* < 0.001

y = 9.658 – 0.0106*x

*R*^2^ = 0.959

*F*(1,148) = 2927.94, *P* < 0.001

y = -6.814 + 0.007528*x

*R*^2^ = 0.943

*F*(1,148) = 1746.79, *P* < 0.001

**C.**

**Figure S8.** Conceptual model of how home climate shapes stomatal morphology and drought response in A. gerardi.


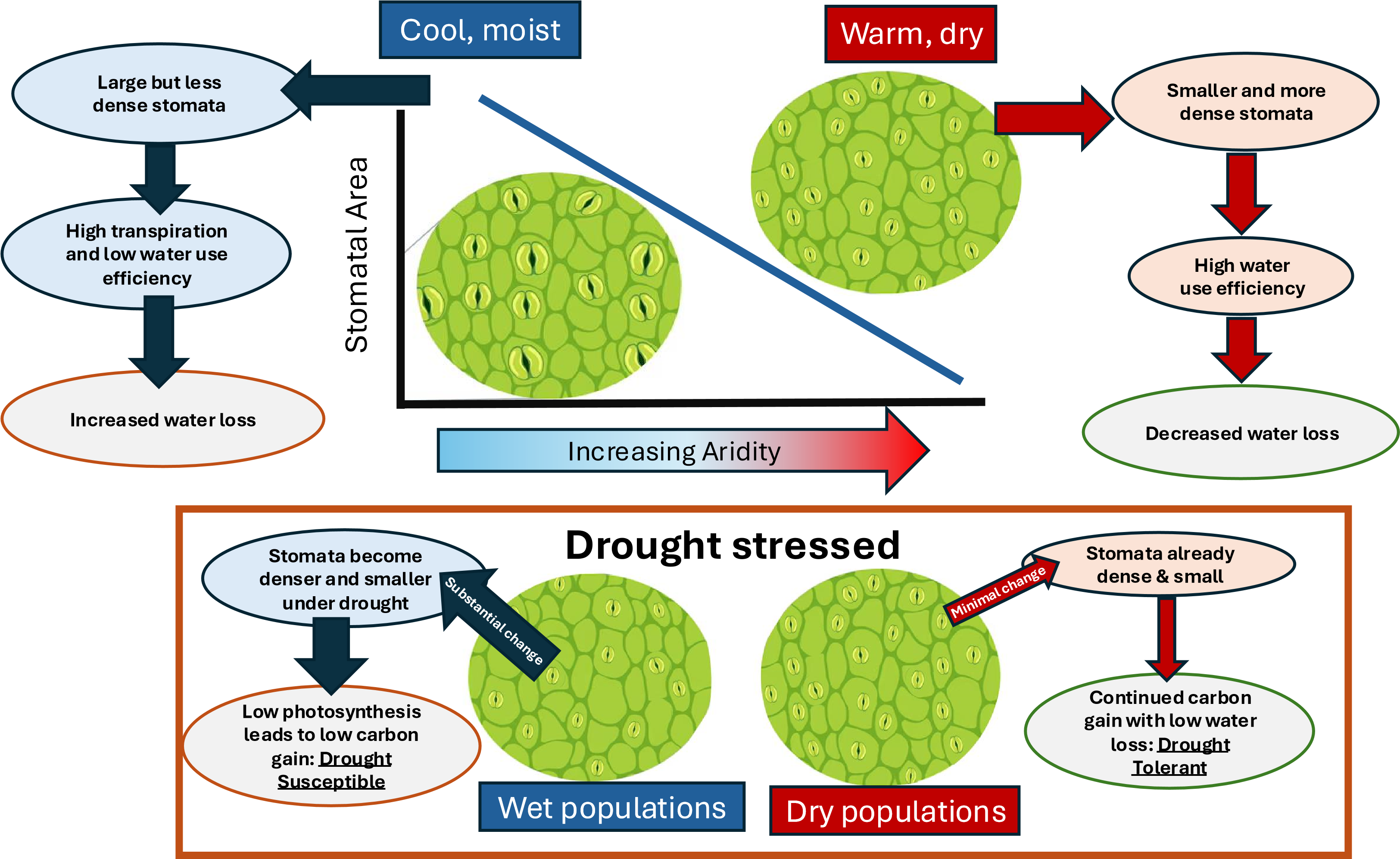


Intraspecific Variation in Stomatal Architecture in *A. gerardi* across Aridity Gradients
